# *In vivo* quantitative high-throughput screening for drug discovery and comparative toxicology

**DOI:** 10.1242/dmm.049863

**Published:** 2023-03-20

**Authors:** Patricia K. Dranchak, Erin Oliphant, Bryan Queme, Laurence Lamy, Yuhong Wang, Ruili Huang, Menghang Xia, Dingyin Tao, James Inglese

**Affiliations:** ^1^ Department of Preclinical Innovation, National Center for Advancing Translational Sciences, National Institutes of Health, Rockville, MD 20850, USA; ^2^ Metabolic Medicine Branch, National Human Genome Research Institute, National Institutes of Health, Bethesda, MD 20817, USA

**Keywords:** Drug discovery, Infectious disease, Genetic disorders, High-throughput screening, Laser cytometry, Model organisms, *C. elegans*, Proteomics

## Abstract

Quantitative high-throughput screening (qHTS) pharmacologically evaluates chemical libraries for therapeutic uses, toxicological risk and, increasingly, for academic probe discovery. Phenotypic high-throughput screening assays interrogate molecular pathways, often relying on cell culture systems, historically less focused on multicellular organisms. *Caenorhabditis elegans* has served as a eukaryotic model organism for human biology by virtue of genetic conservation and experimental tractability. Here, a paradigm enabling *C. elegans* qHTS using 384-well microtiter plate laser-scanning cytometry is described, in which GFP-expressing organisms revealing phenotype-modifying structure–activity relationships guide subsequent life-stage and proteomic analyses, and *Escherichia coli* bacterial ghosts, a non-replicating nutrient source, allow compound exposures over two life cycles, mitigating bacterial overgrowth complications. We demonstrate the method with libraries of anti-infective agents, or substances of toxicological concern. Each was tested in seven-point titration to assess the feasibility of nematode-based *in vivo* qHTS, and examples of follow-up strategies were provided to study organism-based chemotype selectivity and subsequent network perturbations with a physiological impact. We anticipate that this qHTS approach will enable analysis of *C. elegans* orthologous phenotypes of human pathologies to facilitate drug library profiling for a range of therapeutic indications.

## Teaser

**Summary:** We describe an *in vivo* quantitative high-throughput screening strategy with *C. elegans*, designed to leverage and engage experience of the model organism community with screening and drug development resources.

## INTRODUCTION

Quantitative high-throughput screening (qHTS) uses the concentration-response relationship to determine pharmacological properties of drug and investigational agent libraries (Inglese et al., 2006). By integrating *Caenorhabditis elegans*-driven phenotypic models of human disease with current and emerging chemical libraries, the qHTS platform described here will facilitate a critical link in translational research (Hooper and Hmeljak, 2022). As pointed out by Kropp et al. (2021), *C. elegans* has shown promise in rare disease modeling and drug discovery. However, to advance a disease field, a robust approach for the identification of pharmacologic interventions includes reliable access and a means to evaluate molecular modalities revealing novel therapeutic mechanisms (Hasson and Inglese, 2013). The *in vivo* qHTS strategy described herein is designed to leverage and engage experience of the *C. elegans* model organism community with National Center for Advancing Translational Sciences (NCATS) screening and drug development resources.

For over 40 years, the nematode *C. elegans* has helped elucidate the molecular and genetic bases underlying a broad range of biological, developmental and behavioral phenotypes (Brenner, 1974; Sulston and Horvitz, 1977). The organism's short 3-day life cycle, minute size (1.5 mm-long adults), anatomical transparency, ease of *in vitro* cultivation and well-defined genetics are among its distinguishing experimental advantages (Riddle et al., 1997; Thompson et al., 2013). Bacteria serve as a live food source for *C. elegans*, and the worms’ rapid replication with *Escherichia coli* permits the expansion and culturing of thousands of animals. *C. elegans* are primarily self-fertilizing hermaphrodites that can cross with males, providing a highly controlled genetic environment. These characteristics, along with a relatively small ∼20,470 protein-coding genome, 35% of the genes of which have human homologs, have made the roundworm a versatile model for human disease, including a means to interrogate rudimentary organ systems physiology (Kim et al., 2018).

Disease studies in *C. elegans* have included those of the nervous system (neurodegenerative, neuromuscular and axonal neuropathies), as well as metabolic diseases, protein misfolding and aggregation disorders, among others (Caldwell et al., 2020; Kaletta and Hengartner, 2006; Kropp et al., 2021). *C. elegans* have also been used as a host organism for infectious microbes and for anthelmintic development against several parasitic worm species (Campbell, 1985; Ewbank and Zugasti, 2011; Sant'anna et al., 2013). Multiple studies have used *C. elegans* to interrogate the toxicology of potential anthelmintic agents based on viability, lifespan and progression through various life stages. High-throughput screening (HTS) assays tracking these parameters to quantify compound efficacy and potency have been performed mainly using a single concentration treatment paradigm in a 96-well plate format (Breger et al., 2007; Petrascheck et al., 2009; Zhou et al., 2011).

In general, HTS has focused primarily on biochemical and cell-based assay formats, and efforts to incorporate the use of *C. elegans* have been comparatively moderate in scope and number of applications (Gosai et al., 2010; Inglese et al., 2007; O'Reilly et al., 2014). This, in part, can be attributable to growth condition incompatibility with large-scale screening, the amount of compound needed for dosing in traditional treatment paradigms, and labor-intensive handling of the worms (Brenner, 1974; Kwok et al., 2006). However, RNA interference (RNAi) screening in *C. elegans* led to the use of the liquid culture in a 96-well microtiter plate format that was rapidly adapted for small-molecule drug screening (Lehner et al., 2006; Moy et al., 2006). The incorporation of automated liquid handling systems, image acquisition and high-throughput data algorithms for image analysis, and the miniaturization of assays to 384-well and microfluidic formats have increased the efficiency of screening efforts in this whole-organism system (Gosai et al., 2010; Mondal and Ben-Yakar, 2020).

Significant drawbacks in the implementation of HTS with *C. elegans* remain the potential for contamination or inconsistencies introduced from the bacterial food source over multiple-day time courses. Bacterial overgrowth in wells due to metabolically impaired, stressed or dead worms can impact movement and introduce environmental stress to the living worms, or interfere with the data acquisition of specific phenotypes. Additionally, concern that effective compound concentrations can be reduced when using live bacteria due to metabolism or degradation may result in the need to use higher compound concentrations (O'Reilly et al., 2014).

Accordingly, heat-inactivated or antibiotic-treated *E. coli* have been employed to avoid these confounding effects (Gosai et al., 2010; Zheng et al., 2013). In an alternative approach, we utilized *E. coli* ‘ghosts’ that can be prepared in bulk and stored for months to years to serve as a comparative food source for *C. elegans.* Bacterial ghosts (BGs) are cellular membrane envelopes from Gram-negative bacteria generated from the controlled expression of a recombinant PhiX174 lysis gene *E* initially employed for vaccine development and expanded as a vector for adjuvant, antigen and drug delivery (Kudela et al., 2010). However, their use as a potential food source for *C. elegans* had not yet been reported.

The majority of HTS experiments involving *C. elegans* rely on microscopy-based high-content screening (HCS) to measure a phenotypic outcome, often by image analyses. Although HSC is indeed a powerful measurement technology, HCS in this setting generally requires the acquisition of multiple fields to image a given well, accompanied by plate read times that can approach an hour or more. With wide-field imaging devices, a balance of speed and image quality is especially pertinent for fluorescent signals that must have sufficient detection intensity (Buchser et al., 2014). Exceptionally large data files from HCS can present a computationally intensive analysis effort, requiring complex algorithms to recognize and quantify the phenotype.

A sensitive and efficient option for HCS is microtiter plate-based laser-scanning cytometry (LSC), a technology conceptionally comparable to flow cytometry (Zuck et al., 1999). In LSC, cells, particles or more complex objects with intrinsic fluorescence above the background are detected and parametrically evaluated by the size, intensity and fluorescence distribution of select-gated objects (Kamentsky and Kamentsky, 1991). Independent of microtiter plate well density, LSC instrumentation can capture and quantify multiparameter data from individual particles on the fly, gate data to segregate subpopulations within each well across an entire plate, and acquire TIFF images, which can be further analyzed with HCS analysis software, in ∼5–8 min (Auld et al., 2006). LSC is highly adaptable to whole-organism screening when the entire body, an organelle, or a protein of interest is fluorescently labeled. Since its introduction, LSC has enabled a range of ligand-binding and cell-based assays (Auld et al., 2006; Burgess et al., 2020; Clare et al., 2019; Sherman and Pyle, 2013; Zuck et al., 1999).

The generation of large-scale compound library pharmacology constitutes the conceptual basis of the qHTS paradigm (Inglese et al., 2006). When applied to model organism-based phenotypic assays, the approach is enabled at the level of *in vivo* physiology. In this context, qHTS can, for example, categorize physiologically relevant chemical classes for subsequent evaluation in chemo-proteomics-driven network analysis, or support their toxicological assessment for potential human or environmental liabilities. Here, we demonstrate examples of multicellular organism qHTS using *C. elegans* to prioritize pharmacological responses from >600 annotated anti-infective molecules for subsequent bottom-up proteomics analysis (Burns et al., 2021), and in the survey of >800 environmental chemicals studied across *in vitro* assays in the U.S. Department of Health and Human Services Toxicology in the 21st Century (Tox21) program (Xia et al., 2018)*.* The platform is enabled by a novel non-replicating bacterial food source and the multiparametric detection sensitivity and rapid scanning speed of LSC to measure a fluorescent protein-encoded *C. elegans* phenotype (Hobert and Loria, 2006).

## RESULTS

### *E. coli* BGs support *C. elegans* growth

**Fig. 1.**
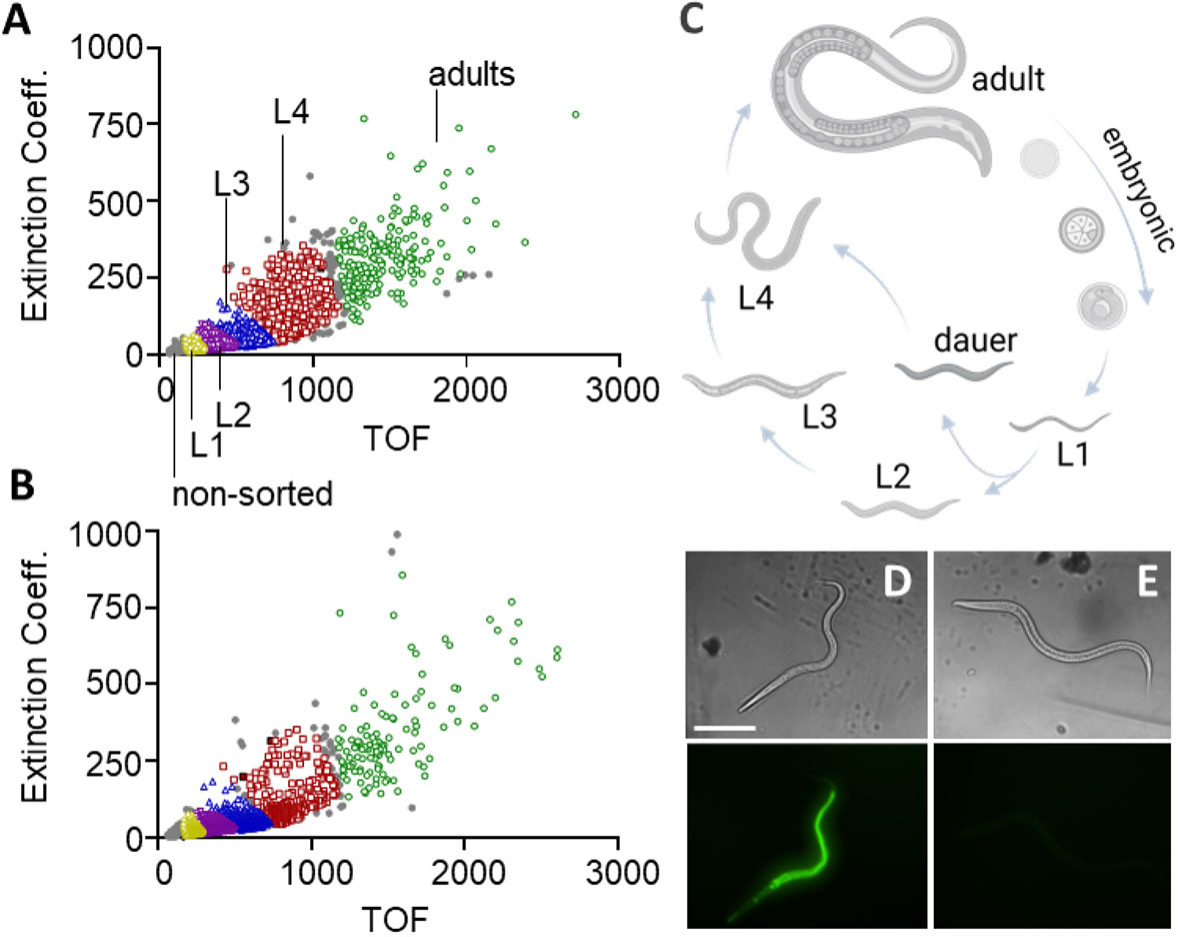
***C. elegans* life-stage analysis on a non-replicating bacterial nutrient.** (A,B) Scatter plots of strain N2 *C. elegans* life stage-sorted worms grown for 5 days on TOP10 bacterial ghosts (BGs) (A) or OP50 live bacteria (B). (C) Life stages were sorted on a COPAS biosorter based on time of flight (TOF) and extinction coefficient, with the various life stages shown as colored symbols and non-sorted objects shown as gray symbols in A and B. (D,E) Representative brightfield (top) and fluorescence (bottom) microscopy images of L3 *C. elegans* strain N2 grown for 5 days on either TOP10 BGs (D) or OP50 live bacteria (E) pre-incubated with DiBAC4(3). Images were collected on an InCell 2200 imager with a 10× objective; brightfield and FITC filters were applied. OP50 live bacteria do not take up DiBAC4(3). Scale bars: 130 μm.

To establish a stable non-replicating *C. elegans* nutrient reservoir suitable for a multi-day qHTS experiment, we investigated the use of *E. coli* BGs. We compared whether worms would actively feed, display normal life stage progression and reproduce when grown on BGs. Microscopic examination of BG-fed worms revealed similar numbers and mobility to a mixed population of worms grown on OP50 live bacteria. Nematode growth medium (NGM) plate-harvested worms analyzed by a COPAS FP-500 biosorter demonstrated an equivalent life-state distribution whether grown on live bacteria or BGs (Fig. 1A−C).

DiBAC4(3), a bis-oxonol membrane potential-sensitive dye selectively taken up by depolarized, nonviable bacteria, demonstrated that BGs filled the digestive tract of the *C. elegans* (Fig. 1D,E). N2 worms were grown on OP50 live bacteria or TOP10 BGs with or without DiBAC4(3) dye for 5 days at an ambient temperature of ∼22°C spanning approximately two full life cycles. Fluorescence was detected, by COPAS biosorter analysis, only in the BG-fed worms as only nonviable worms can take up the dye (Fig. S1), with fluorescence increasing in intensity from L1 larvae to adults. These experiments indicate that *C. elegans* can feed on BGs and that this novel food source sustains normal worm development and brood size.

### 384-well LSC *C. elegans* viability assay

**Fig. 2.**
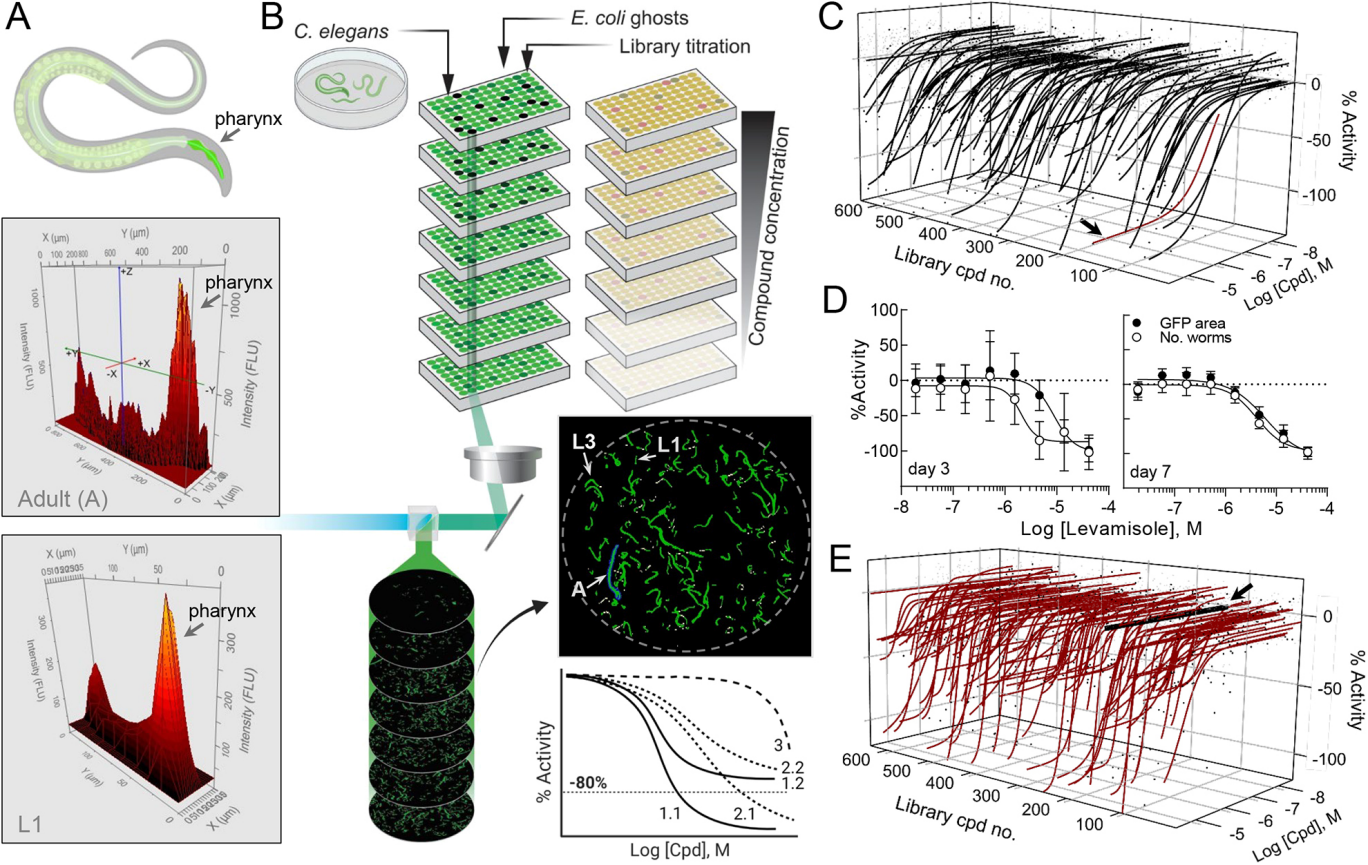
***C. elegans* laser-scanning cytometry (LSC) quantitative high-throughput screening (qHTS).** (A) Area (*x*-*y*)–intensity (*z*) plots for a representative adult (A) and L1 *C. elegans* life stage, in which the pharynx is clearly visible as the plot feature with the greatest GFP intensity. (B) Representation of the *C. elegans* qHTS process, highlighting the use of 384-well inter-plate compound titrations, LSC output indicating the relative sizes of L1, L3 and adult worms, and the general curve classification (CC) scheme for CC=1 (solid lines), CC=2 (dotted lines) and CC=3 (dashed line) for dose–response curves (DRCs). (C) Three-axis plot of DRC profiles for GFP area of worms on day 7 for the 643 compounds in the NCATS Anti-Infectives collection; 4501 dose–response values are displayed in black dots. The 83 compounds with a decreased GFP area are plotted as black curves. Black arrow indicates the non-control ivermectin library sample DRC (red curve). (D) DRCs for the levamisole screening control at day 3 [GFP area (filled circles), 9.37±0.93 μM; number of worms (open circles), 5.96±2.40 μM] and day 7 (GFP area, 7.99±1.44 μM; number of worms, 5.74±0.91 μM). Error bars indicate s.e.m. (*n*=21). Data were normalized to 41.7 μM levamisole as −100% activity, and half-maximal effective concentration (EC_50_) values were determined from logistic fits (GraphPad Prism), averaged from 21 replicates. (E) Compound library effect on HEK293 cell viability determined with Cell TiterGlo. The 643 compounds from the NCATS Anti-Infectives collection above were screened and 4501 dose-response values are shown as black dots. The 147 compounds that were cytotoxic to the human cells are shown as red curves. The black arrow indicates the non-control ivermectin library sample DRC (black line). Curves in three-axis plots were fit using a four-parameter logistic regression software (see Materials and Methods for details).

To verify BGs as a *C. elegans* growth medium in 384-well microtiter plate format, the viability of various developmental stages of *C. elegans* strain PE254, which ubiquitously express GFP, was assayed while the worms were grown on BG media or OP50 live bacteria with LSC. The viability of GFP-expressing worms was quantified based on two parameters: (1) total GFP area (µm^2^) of gated objects, and (2) total gated GFP object number (‘number of worms’) derived from integrated objects (Fig. 2A), under either dimethyl sulfoxide (DMSO) or 50 µM levamisole anthelmintic control treatment in 384-well format. Worms grown on live bacteria did not approach significance for HTS based on the Z-factor (Z′), a statistical measure to determine whether the assay signal is reliably above background (Zhang et al., 1999), for any life stage or time point investigated. Worms grown on BGs demonstrated Z′ above the conventional HTS threshold of 0.5 for early larval stages, while later larval stages and adults had Z′ above 0.3, which supports qHTS feasibility (Fig. S2, Table S1).

*C. elegans* were further assayed across a titration of three anthelmintic drugs – ivermectin, albendazole or levamisole – over 7 days. Each displayed differential life stage sensitivity to the drugs (Fig. S3). In general, worms grown on live bacteria exhibited more microtiter plate variability than those grown on BGs (Fig. S2B, Fig. S3A,B). Additionally, early larval-stage worms appeared to progress through their life cycle and reproduce quicker on BGs than on live bacteria. Nonetheless, accounting for growth rates, the relative pharmacotoxicity of the drugs was similar in worms whether grown on live bacteria or BGs (Fig. S2C−F).

Ivermectin, the most potent anthelmintic, displayed an ∼1 nM half-maximal effective concentration (EC_50_) for all life stages grown on BGs by day 1 (Table S2A). Albendazole, a microtubule poison that slows growth and disrupts the larval cuticle and oocyte development, has been reported to be toxic (EC_50_>10 µM) at all life stages post 7-day treatment (Sant'anna et al., 2013), and this is consistent with our own observations for worms grown on live bacteria, although the drug was slightly more potent (EC_50_=500 nM to 4 µM by day 7 for all life stages), having minimal toxicity earlier in the time course. Worms grown on BGs presented significant toxicity as early as day 1 for L4 and adult worms, and by day 2 for most life stages (Table S2B), correlating with the more rapid life cycle progression and egg laying observed on BGs. Levamisole gave similar EC_50_ values in the low-micromolar range for all life stages grown on both media across the 7-day time course (Table S2C).

### *C. elegans* LSC qHTS

An overview of the phenotypic qHTS configuration utilizing *C. elegans* grown on an *E. coli* BG nutrient source is shown in Fig. 2B. Here, a library of 643 agents with a variety of anti-infective mechanisms was prepared as a seven-point inter-plate titration series in 384-well assay plates, to which nutrients and *C. elegans* were subsequently added. *C. elegans* viability was assessed according to worm number and total GFP area per well, respectively, on day 3 (one life cycle) and day 7 (more than two full life cycles) post treatment. As expected, both parameters gave a greater signal-to-background ratio and improved assay statistics on day 7 compared to day 3, although only the GFP area approached a HTS significant Z′ of 0.5 (Zhang et al., 1999) on day 3 (Table 1). EC_50_ values determined from both parametric outputs for levamisole, the intra-plate screening control, were similar on day 7 and correlated well with published values (Fig. 2D) (Qian et al., 2008). Additionally, three anthelmintic controls (ivermectin, levamisole and albendazole) were included as inter-plate controls on one assay plate and read every 24 h over the 7-day time course. EC_50_ values, although slightly higher than previously observed (Table S2), demonstrated activity at the expected time points (Table 2; Fig. S3D). In parallel, an HEK293 cell viability assay was conducted to estimate the compound toxicity window between human cells and the nematode for all library compounds (Fig. 2E; Fig. S4 and Table S3), in which ivermectin (black arrows, Fig. 2C,E) illustrates an ideal species-selective toxicity window.

**Table 1.**
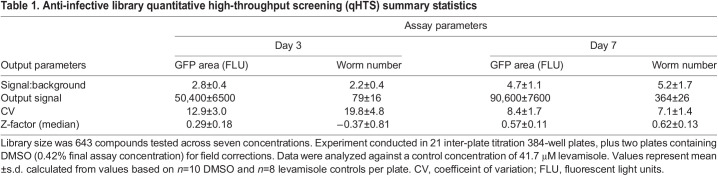
Anti-infective library quantitative high-throughput screening (qHTS) summary statistics

**Table 2.**
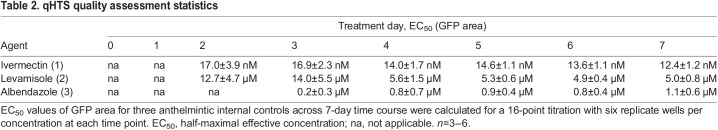
qHTS quality assessment statistics

Analysis of GFP area on day 7 yielded 83 compounds that inhibited worm viability based on curve classifications of −1.1 to −3, with EC_50_ values ranging from 10 nM to >50 µM (Tables S3 and S4). As well as ivermectin, two other anthelmintics, pyrvinium pamoate and L-tetramisole (levamisole), were identified as active, whereas the fourth antiparasitic compound in the library, praziquantel, was not detected [curve classificiation (CC)=4]. Active compounds were selected for follow-up analysis based on qHTS curve classification assignment (Inglese et al., 2006), ranking first for activity on days 3 and 7, followed by compounds with moderate day 3 GFP area activity, and having low to no toxicity in the human cell line counter qHTS.

### Anti-infective follow-up studies

**Fig. 3.**
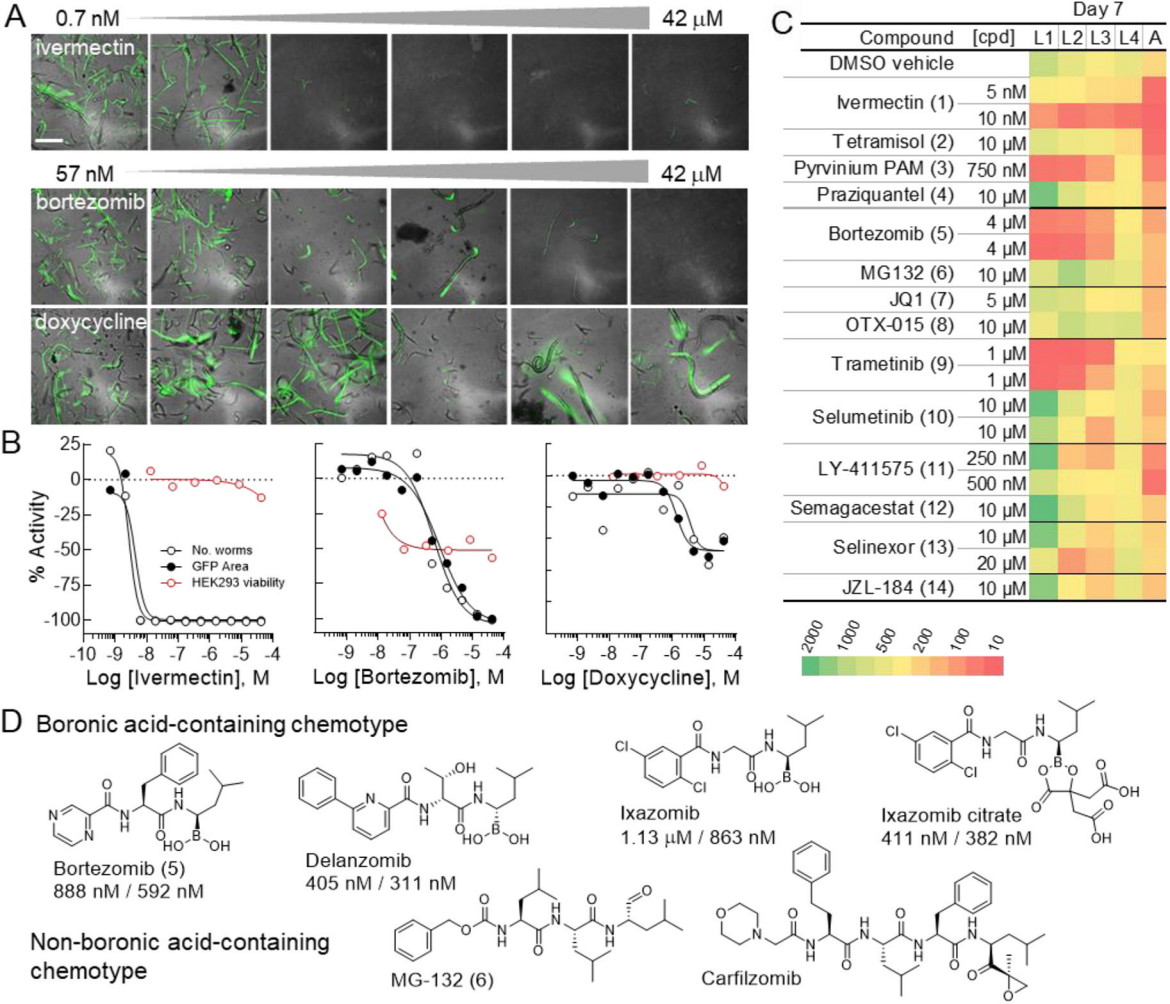
**LSC–microscopy correlation and life-stage analysis.** (A) Representative merged GFP and brightfield images obtained from the InCell 2200 across the active concentration range for the anthelmintic, ivermectin (top), the proteasome inhibitor, bortezomib (middle), and the antibiotic doxycycline (bottom). Scale bars: 345 μm. (B) Re-test 11-point dose–response curves for representative active molecules from three mode-of-action classes identified in the primary qHTS and general cellular toxicity in mammalian cells (HEK293 cell line, red open circles). The two laser cytometer output parameters (GFP area, black filled circles; worm number, black open circles) measured on day 7 of the qHTS time course are plotted as data normalized to 41.7 μM levamisole screening control as −100% activity. Curves were fit in GraphPad Prism. (C) Day 7 life-stage distribution for larval stages L1–L4 and adult (A) as determined by COPAS flow cytometry. Worms were plated and treated as a mixed population of 2000 worms/well at day 0. The scale ranges from >15,000 (dark green) to <10 (red) animals. Compounds selected for evaluation on impact of life-stage viability: 1–4, antihelminth agents; 5 and 6, proteasome inhibitors; 7 and 8, bromodomain inhibitors; 9 and 10, MEK inhibitors; 11 and 12, γ-secretase inhibitors; 13, nuclear protein export inhibitor; 14, monoacylglycerol lipase inhibitor. (D) Boronic acid-based proteasome inhibitors and qHTS potency based on GFP area and worm number, respectively, and inactive non-boronic acid class of proteasome inhibitors represented in the library.

Ninety-six compounds were selected for follow-up analysis based on the prioritization scheme above, 78 of which demonstrated activity on the parameter of GFP area on day 7 in the initial screen (Table S4). These compounds were tested across a 7-day time course in an 11-point intra-plate titration, analyzed by LSC, and worm numbers were compared with high-content imaging (Fig. 3A). Both LSC parameters, GFP area and worm number, were analyzed on day 7 with acceptable assay quality metrics, and the levamisole assay control demonstrated consistent EC_50_ values between the two measured parameters and those values calculated in the initial screen (Fig. S5). Of the 96 compounds analyzed, 73 reconfirmed activities on both GFP area and worm number at day 7, 19 were inactive and four compounds were selectively active with one parameter but not the other (Table S5). Over half the 96 molecules analyzed shared a common annotated mechanism of action (MOA) with at least one other compound in the follow-up subset.

The three most active classes of molecules based on efficacy and potency included the anti-nematodal agents (three of three identified), proteasome inhibitors (four of ten found active in the primary screen; all four reconfirmed activity), and bromodomain inhibitors (four of six were identified as primary qHTS actives and reconfirmed activity). Additional compounds demonstrating effective cytotoxicity on *C. elegans* included an antifungal agent and one antimalarial agent, two MEK-1/2 inhibitors, both monoacylglycerol lipase inhibitors in the collection, and the two nuclear export inhibitors in the library (Fig. S6A). Also, five compounds listed as anticancer/antioxidant agents, two C-C chemokine receptor antagonists and three DNA topoisomerase II inhibitors were found to have moderate activity. Among each of these classes were several inactive members, suggesting species-related chemotype selectivity or potential off-target effects accounting for the observed activity (Fig. S6B, Table S3).

Two of the largest library categories included antibacterial/antimicrobial and antivirals. Several molecules from both were selected for follow-up where differential activity was observed within the respective MOA class; several inactive molecules were also included (Tables S3 and S4). Two proteolytic secretase inhibitors demonstrated selective activity between gamma- and beta-specific chemotypes; deubiquitinase inhibitors and apoptosis activators were two other classes with variable activity in the *C. elegans* assay (Fig. S6C). Finally, 41 compounds had individual annotated MOAs within the follow-up subset, of which tyrphostin A9, auranofin and oligomycin A had the best activity (Fig. S6D).

### Microscopy–laser cytometry correspondence

To confirm the correlation between LSC-derived dose–response curves (DRCs) and microscopy-based analysis, follow-up plates were imaged on an InCell 2200 on day 7 (Fig. 3A). For each molecule, representative images from the active concentration range were visually inspected for worm number and health based on GFP and brightfield views. The potency of the various molecules appears to be highly correlated between the LSC- and microscopy-based imaging technologies, where efficacies based on the LSC DRCs across classes of MOA were also confirmed in the high-content imaging, as visualized by the differential survival of worms under specific compound treatments (Fig. 3A,B). Additionally, although the worms appeared to be overlapping and clustered in the high-content images, which can make segmentation difficult with HCS algorithms, the number of worms calculated on the fly in LSC with the optimized gating parameters accurately enumerated worms in each well, providing results very similar to the curves plotted from total GFP worm area (Fig. S5).

### Life-stage flow cytometry analysis

For six mechanistic classes, we analyzed representative chemotypes (Fig. S7) at their ∼EC_75-80_ for effect on life stage viability after a 7-day treatment using the COPAS biosorter to enumerate organism subpopulations, summarized in the Fig. 3C heat map. Among the four re-tested anti-nematodal agents, differences in larval and adult populations were observed. We presume that the dramatic treatment effects at 5 nM versus 10 nM for ivermectin reflect the very steep dose response of this drug as mirrored in the follow-up DRC pharmacology (Fig. 3B), whereas the more graded hill slope observed (Fig. S6A) for tetramisol (compound 2) and pyrvinium pamoate (compound 3) permitted a more straightforward re-test concentration determination. As expected, the trematode parasitological agent, praziquantel (compound 4) had no effect on any *C. elegans* life stage, in agreement with the qHTS results (Table S3).

Selectivity was observed among chemotypes (Fig. S7) targeting the same proteins or pathways. For example, as seen in Fig. 3C, the proteasome inhibitors, bortezomib (compound 5) and MG123 (compound 6), and the MEK inhibitors trametinib (compound 9) and selumetinib (compound 10), displayed potent larval-stage depletion or no discernable effect, respectively. Here, such chemotype-associated (e.g. Fig. 3D) activity differences are not unexpected due to low sequence conservation between the human and nematode target proteins, although species-specific drug uptake and metabolism can also account for diminished or absence of compound activity.

### Label-free proteomics quantitation analysis

**Fig. 4.**
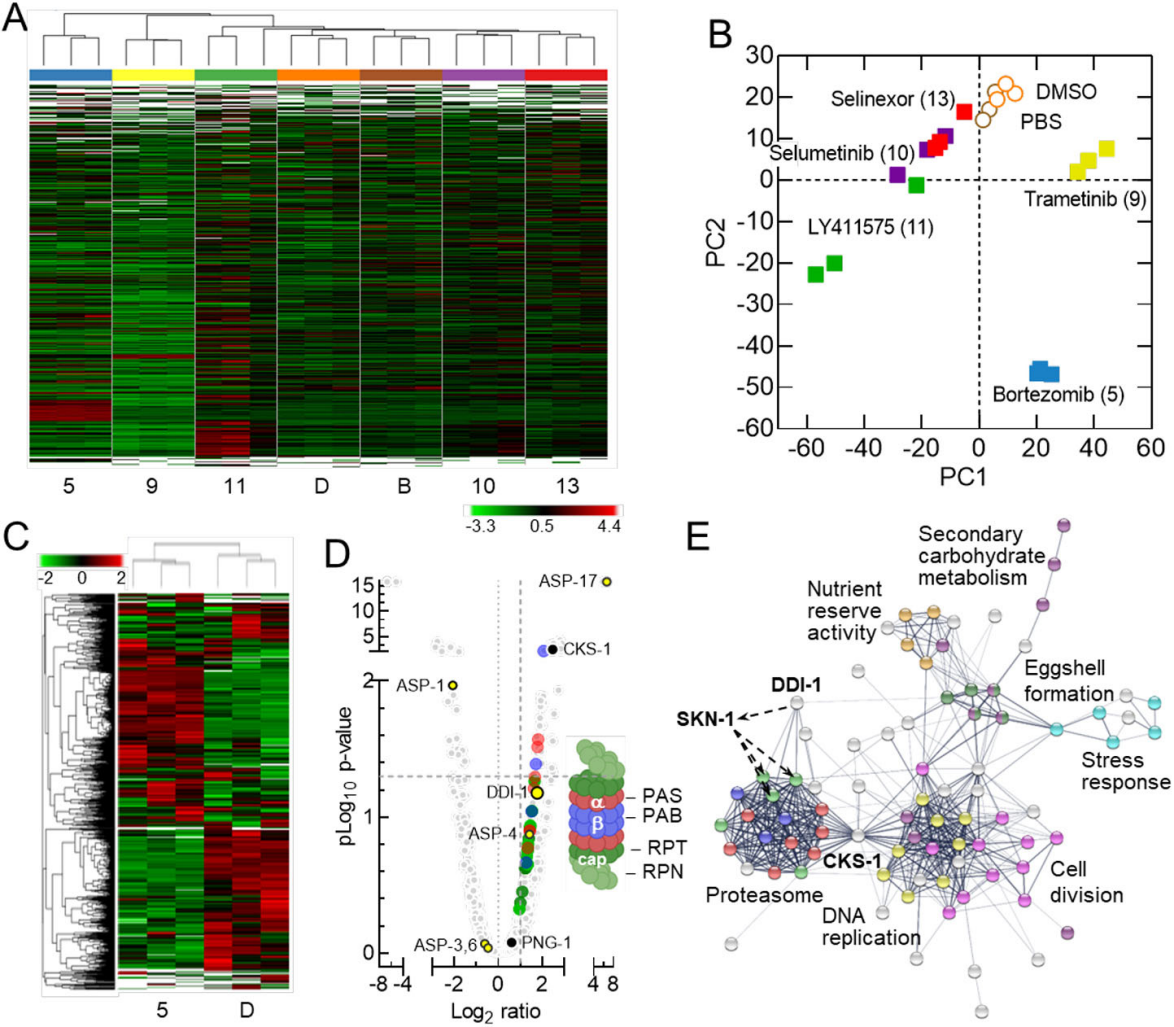
**Proteomics analysis of drug-treated *C. elegans*.** (A) Hierarchical cluster analysis using normalized protein abundance derived from a bottom-up quantitative mass spectroscopy-based proteomics analysis of *C. elegans* following a 7-day treatment with the indicated compounds or controls (D, DMSO; B, buffer). Label-free quantitation was applied for each group comparison with three biological replicates for each compound treatment and control condition. (B) Principal component analysis from 2645 protein groups identified from 37,992 peptides for experimental triplicate determinations. (C) Hierarchical cluster analysis as in A for proteome changes induced by bortezomib versus DMSO vehicle control. (D) Volcano plot showing the statistical significance (pLog_10_ *P*-value) versus fold change (Log_2_ ratio) for 2563 *C. elegans* proteins (light gray circles) after 7 days of exposure to 4 µM bortezomib (compound 5) versus DMSO control. The 2-fold abundance increases (right of the vertical dashed line) are highlighted for proteins associated with the α, β and cap subunits (red, purple and green circles, respectively) that comprise the proteasome (right inset). Open circles are aspartyl proteases; filled circles are proteasome-associated network proteins. The horizontal dashed line indicates the *P*-value threshold of 0.05. (E) A protein–protein interaction network constructed with STRING v.11.5 using upregulated proteins from the volcano plot in D. Coloring of the proteasome node correspond to those used in D. The presence of SKN-1 is inferred.

The annotated genome and pathway databases of *C. elegans* can inform the interpretation of phenotypic analysis pertaining to mechanism of action studies (Davis et al., 2022). To investigate this, we prepared digested peptides from protein extracted from ∼2000 treated or control worms in biological triplicates, based on life-stage analysis (Fig. 3C), and subsequently analyzed the samples by high-performance liquid chromatography–tandem mass spectrometry (HPLC-MS/MS) (Burns et al., 2021). In brief, 2645 protein groups and 37,992 peptides were identified from seven groups of samples (Table S6). Hierarchical clustering of the identified protein groups revealed clusters of upregulated and downregulated proteins (Fig. 4A), where molecules affecting life-stage progression or number (compounds 5, 9, and 11) were clearly separate from vehicle controls (DMSO or PBS) and inactive chemotypes (compounds 10 and 13) for targets of active compounds. Principal component analysis from these data demonstrated a generally strong correlation of replicate treatments, especially for trametinib, bortezomib and vehicle controls (Fig. 4B).

As an example of deeper phenotype interrogation, we examined the well-replicated bortezomib (compound 5)-induced proteome perturbations in finer detail. A direct comparative hierarchical clustering of the identified protein groups from bortezomib and DMSO treatments allows the identification of proteins with statistically significant changes in abundance. From these proteomic changes, the cellular consequence of proteasome inhibition was used to further elucidate additional relevant processes (Fig. 4C; Tables S7 and S8). These included, for proteins observed to increase in abundance by 2-fold or greater (Fig. 4D), all seven subunits comprising the α (PAS) and four of seven β (PBS) subunits of the 20S core particle, and nine of the 15 cap subunit proteins (RPT and RPN) that make up the 19S regulatory particle of the 26S proteasome (Davy et al., 2001). A Search Tool for the Retrieval of Interacting Genes/Proteins (STRING) network analysis focusing on upregulated proteins clearly categorized the proteasome and additional causally linked cellular processes, particularly DNA replication and cell division through the significantly upregulated cyclin-dependent protein kinase regulatory subunit CDC28 ortholog, CKS-1 (Fig. 4E) (Szklarczyk et al., 2019). Several aspartyl proteases were observed to be upregulated as well. Among these, DDI-1 is responsible for the N-terminal processing of the transcription factor SKN-1, required for proteasome gene expression (Kropp et al., 2021). Although SKN-1 was not detected, but rather inferred here, PNG-1, critical to the N-glycosyl asparagine editing of SKN-1, generating the aspartyl SNK-1 precursor DDI-1, substrate was detected in the *C. elegans* proteome (Fig. 4D; Tables S7 and S8).

The translational upregulation and/or half-life stability of drug-targeted proteins or complexes, and associated networks demonstrate the organism's complex drug-mediated loss-of-function response. The subtle perturbations of associated processes are revealed and illustrated from a STRING network analysis exemplifying means to probe the physiological basis of phenotype-altering chemical agents (Kale and Moore, 2012; Szklarczyk et al., 2019).

### *In vivo* qHTS toxicology

**Fig. 5.**
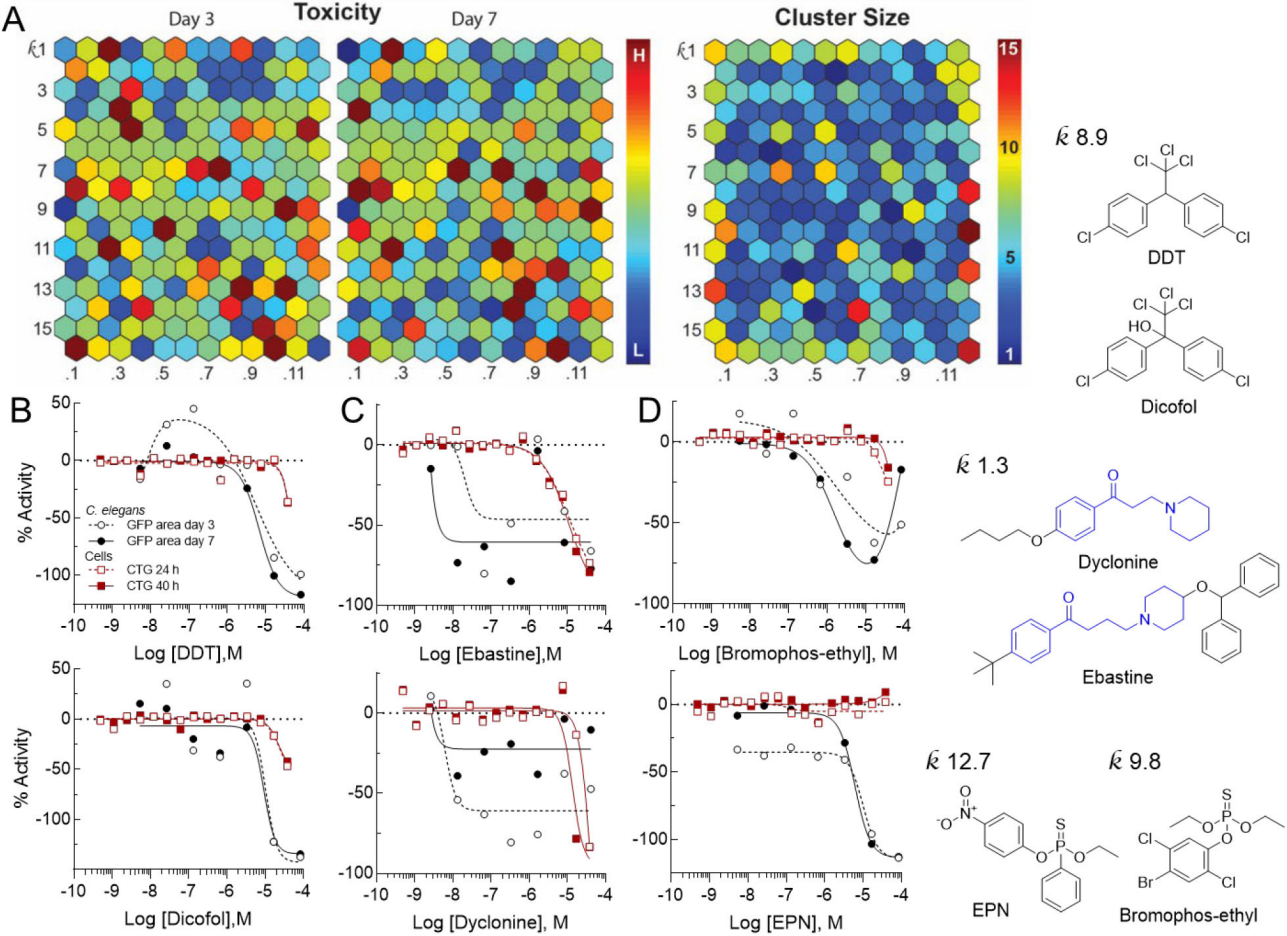
**Analysis of Tox21 sub-library *C. elegans* qHTS output.** (A) Structural–toxicity relationships as evaluated using the self-organizing maps algorithm. In these heat maps, each hexagon represents a cluster of structurally similar compounds. Clusters enriched with potent active compounds (compared to the library average) are shown in red; blue clusters are deficient of active compounds. Color scale values represent the log *P*-value for each cluster that measures the statistical significance of enrichment from Student's *t*-test. (B–D) Representative dose–response curves plotted with the laser cytometer output parameter *C. elegans* GFP area (day 3, black open circles; day 7, black filled circles) with data normalized to 41.7 µM levamisole screening control as −100% activity, and for HEK293 cell viability data from the Tox21 program (24 h, red open squares; 40 h, red filled squares) for several enriched clusters and chemotypes – k8.9, endocrine disruptors, DDT and dicofol (B); k1.3, piperidinyl alkylphenones (blue substructure), dyclonine and ebastine (C); and organophosphates, EPN and bromophos-ethyl (D) – distributed among different clusters.

Finally, the pharmacological output of *C. elegans*-based qHTS demonstrated use in a comparative toxicology setting in the evaluation of >800 environmental chemical agents with potential toxicity identified from the Tox21 effort (Table S9). The qHTS was performed with similar statistics as observed for the previous anti-infectives library screen (Table 3), and, in this case, we used total GFP area to score the activity measured on days 3 and 7 post chemical exposure. These 887 compounds were clustered based on a structural similarity approximation using the self-organizing maps (SOM) algorithm (Kohonen, 2006), yielding 192 k-clusters comprising between 1 and 15 members (Fig. 5A; Table S10). Of these clusters, 20 were significantly enriched with compounds that inhibited *C. elegans* growth or survival, with the ivermectin cluster k16.1 displaying the most potent response (Fig. S8). Additional instances of clusters displaying potent activity include k8.9, represented by dichlorodiphenyltrichloroethane (DDT) and its analogs, k1.3, containing a range of pharmacologically active substances with a shared piperidinyl alkylphenone substructure, and the organophosphates, irreversible acetylcholinesterase inhibitors (Fig. 5B−D). This latter category of 20 phosphates, thiophosphates and phosphonothioate O-ethyl O-(4-nitrophenyl) phenylphosphonothioate (EPN) were distributed among 13 k-clusters, illustrating a limitation of the SOM algorithm in chemotype identification. This is likely because the clustering algorithm, based on whole-molecule similarity, does not prioritize substructure features such as a phosphate group when defining a cluster (Fig. S9). In addition to these compounds, 15 potential mitochondrial toxins, such as chlorfenapyr (k7.8) and rotenone (k12.2), were identified from a previous *C. elegans* study of the Tox21 program (Xia et al., 2018) and were also identified in the current *C. elegans* qHTS assay. As shown in Table S11, there is a very good correlation between *C. elegans* qHTS and previous ATP and larval growth assays, further validating *C. elegans* qHTS as a large-scale quantitative profiling method to evaluate *in vivo* compound toxicity.

**Table 3.**
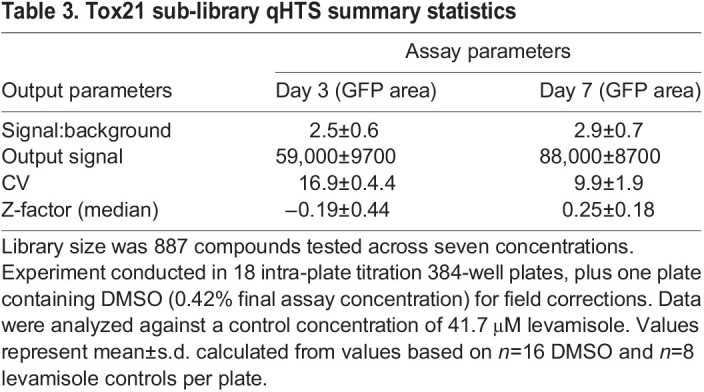
Tox21 sub-library qHTS summary statistics

## DISCUSSION

Here, we describe qHTS employing a multicellular organism evaluated over several generations. Building on prior work reporting examples using *C. elegans* in chemical library screens (Gosai et al., 2010; Iyer et al., 2019; Knox et al., 2021; Ye et al., 2014), our study attempts to expand the versatility of *C. elegans* as a qHTS-compatible model organism. Towards this end, *E. coli* BGs were employed as a non-replicating nutrient for the multi-day screen, providing a stable, consistent *C. elegans* food source that can be prepared, characterized and stored for years. BGs eliminate possible variability that can accompany the preparation of heat-inactivated or antibiotic-treated *E. coli*, and possible environmental stress of live bacteria on relatively low worm densities (approximately ten worms/well) used in qHTS (Fig. S2). Importantly for qHTS, the use of BGs as a ‘reagent’ simplifies the workflow. The Elysis vector used to produce BGs has been deposited in Addgene (#176891) to facilitate community evaluation and use of this approach.

As a numerical detection output, LSC provided efficient fluorescent signal detection from *C. elegans* expressing GFP in 384-well microtiter plate format, enabling the construction of qHTS dose–response curves. The resulting dose–response curves enabled more informed prioritization for follow-up chemotype cluster re-testing, and life-stage and proteomics analyses. There are certain constraints in applying qHTS to *C. elegans*. For example, the animal number and size distribution described here preclude the surface area/volume limitations of 1536-well plates routinely used in large-scale chemical library qHTS (Inglese et al., 2006). Further, scale-up of synchronized *C. elegans* populations suitable for very large library qHTS would present logistical challenges. However, as demonstrated in this study, *C. elegans* qHTS is ideally suited for use with increasingly available high-impact chemical libraries of a moderate size, such as approved drugs, protein kinase inhibitors, academic consortium libraries or environmental chemical collections (Brown et al., 2011; Drewry et al., 2014; Huang et al., 2011; Kearney et al., 2018; Ngan et al., 2019).

LSC was highly effective in scoring worm populations for the construction of DRCs to permit rapid sorting via CC values for subsequent re-testing (Tables S3−S5). Although not necessary in the current study, additional LSC object parameters, such as aspect ratio and Gaussian distribution of fluorescent signal, can categorize worms based on shape and morphology, e.g. the natural sinusoidal versus curled phenotype initially observed upon treatment with paralytic agents such as levamisole and albendazole (Lycke et al., 2013; Sant'anna et al., 2013). In addition to phenotypic classifications possible from fluorescently tagged proteins or anatomical regions such as the gut, body wall, pharynx or neurons, the three-fluorescent channel capability of LSC would, in principle, enable colocalization and multiplexed assay formats (Auld et al., 2006). For example, metabolic incorporation of TAMRA-dATP into genomic DNA or ‘cell painting’ could facilitate a range of phenotypic profiles in *C. elegans* (Gustafsdottir et al., 2013; Schreier et al., 2022). Although we conducted the current study with LSC, using automated microscopy as a primary or LSC-coupled output is certainly feasible (Gosai et al., 2010). However, where appropriate, LSC allows a significantly faster plate read time, enumeration of all well objects and on-the-fly analysis. Microscopy is not as rapid, often requires organism paralysis steps for imaging, fewer objects are quantified, and HCS algorithm development for analysis can be involved depending on the parameters and output desired. However, microscopy clearly can provide a more detailed and intricate phenotype analysis than would be possible with LSC.

In this proof-of-principle study, collectively, >1500 compounds were pharmacologically evaluated for an anthelminthic effect on BG-fed *C. elegans* in qHTS format over a 7-day time course using an LSC readout. From the anti-infectives library, three anti-nematodal agents (Fig. 3, compounds 1–3; Fig. S6A) were identified through high-quality dose–response CCs, thus serving as blinded internal controls. A fourth anti-parasitic compound, praziquantel (compound 4; Fig. S7), administered for trematodes and cestodes infections, although with no reported nematode efficacy (Greenberg, 2005), was not identified as active, further demonstrating assay selectivity.

The chemotype-dependent species selectivity demonstrated in this study highlights the importance of including a structurally diverse selection for each mechanistic drug class to best leverage the power of *C. elegans* as a potential phenolog of human disease in chemical library screening. For example, proteasome and bromodomain (BRD) inhibitors were identified as molecular classes with potential anthelmintic properties. For these, four of 11 proteasome and three of seven BRD inhibitors within each class were identified as having high-quality inhibition curves, which were reconfirmed in follow-up analysis (Fig. S6). Among the 11 proteasome inhibitors in the library, only the four boronic acid-containing chemotypes were active (Fig. 3D); the other proteasome inhibitor classes (epoxides, aldehydes, and nitro sulfonic and vinyl amides; Fig. S10A) were not identified as active in the primary screen, illuminating the significant species differences reflected in either the mammalian versus *C. elegans* proteasome sequence or the bioavailability of the chemotype in this organism. For the BRD inhibitors, the differential activity observed among the chemotypes (Fig. S10B,C) is not as clear-cut as with the boronic acids, potentially reflecting various mechanisms, including off-target activities.

The primary screening sensitivity of qHTS may be particularly advantageous when assessing drugs and investigational agents in *C. elegans* for which potency and efficacy are not optimized for this organism. For example, the monoacylglycerol lipase (MAGL) inhibitor, JZL-184, and nuclear export protein inhibitor analogs, selinexor and verdinexor, were captured by qHTS despite having low potency and efficacy on *C. elegans* viability in the primary seven-point screen (Table S3); re-testing in 11-point titration confirmed the observation (Table S5) that they are moderately active in *C. elegans* compared to the human cell line (Fig. S6A). In the case of JZL-184, whether the effect on *C. elegans* viability is promoted by MAGL inhibition or off-target activity on other serine hydrolases such as fatty acid amide hydrolase (FAAH) remains to be determined (Long et al., 2009). However, given the dependence of cholesterol trafficking on endocannabinoid metabolism and signaling, the observed result is consistent with impaired *C. elegans* development (Baggelaar et al., 2018; Galles et al., 2018).

Not surprisingly, reactive or promiscuously acting compounds showed effects on *C. elegans* viability. Here, molecules including pristimerin, tyrphostin A9, auranofin and oligomycin A displayed potent and/or efficacious toxicity often, but not always, with concomitant activity on the human cell line control (Fig. S6B,D), suggesting an application of this assay system in chemical toxicological assessment studies in which detection of adverse effects requires a more sophisticated *in vivo* model than is possible with cell culture systems.

The pharmacological output of qHTS is well aligned with subsequent omics-based analysis. For example, here using mass spectrometry (MS)-directed proteomics, we interrogate the action of proteasome inhibitors on networks tied to this protein complex and its associated cellular processes (Fig. 4E). The rapid deciphering of a drug or toxic response to an underlying mechanism or potential target protein can substantially help advance the search for and development of new therapeutics and potentially flag accompanying liabilities. We anticipate that the platform described here will further integrate the use of *C. elegans* orthologous phenotypic models of human disease for pre-clinical drug discovery or repurposing, and in the assessment of potential environmental and human toxins (Golden, 2017; Kim et al., 2018; McGary et al., 2010).

## MATERIALS AND METHODS

### *C. elegans* culture

*C. elegans* wild-type strain N2 Bristol isolate and strain PE254, feIs4 [*sur-5p*::luciferase::GFP+*rol-6(su1006)*] (Lagido et al., 2009, 2008; McLaggan et al., 2012), were purchased from the *Caenorhabditis* Genetics Center (University of Minnesota) and grown according to Stiernagle (2006). Frozen aliquots were thawed as needed onto fresh 10 cm NGM plates (Teknova) seeded with OP50 *E. coli* grown to an optical density at 600 nm (OD_600_) >0.6. *C. elegans* were maintained at ambient temperature (20–24°C), and their growth was monitored with visual inspection under a 10× microscope. Worms were expanded to new NGM plates with sterile chunking technique, and stock cultures of worms were maintained with frozen aliquots of each strain prepared from freshly starved plates of mixed populations of worms in 16.5% glycerol (v/v) in 1× S basal buffer [5.85 g/l NaCl, 1 g/l K_2_HPO_4_, 6 g/l KH_2_PO_4_, sterilized by autoclaving, followed by addition of 1 ml cholesterol (5 mg/ml in ethanol)], and stored at −80°C. Cultures were monitored for contamination and *C. elegans* health with microscopic inspection (Saul et al., 2021).

### Compounds and anti-infectives library preparation

Levamisole, ivermectin and albendazole were purchased from Sigma-Aldrich. Stock solutions of all compounds were prepared at 10 mM in DMSO, and ivermectin was diluted to a 10 µM working dilution in DMSO. The NCATS Anti-Infectives Collection is a directed assembly of 643 compounds that have demonstrated activity against viral, bacterial or eukaryotic pathogens. Of these, 284 (38%) are approved for clinical use in the United States, 32 (4%) were previously marketed for use in the United States, and 62 (8%) are used clinically outside of the United States. Nearly 90% of the compounds have annotated mechanisms of action against biological pathogens, facilitating interrogation of known antimicrobial targets. DMSO stock solutions of were arrayed in Echo-qualified 384-well cyclic olefin copolymer plates (Labcyte) in qHTS inter-plate dilution with seven concentrations, 1:5 dilution, resulting in a final concentration range of 2.5–20 mM high concentration titrated to 160 nM to 1.28 µM low concentration (Yasgar et al., 2008). ‘Assay-ready’ plates were prepared in 384-well uClear microtiter plates (Greiner BioOne), with compounds acoustically dispensed at 250 nl per well with an Echo 555 in the above inter-plate titration array. Two plates with DMSO added across the compound field at the same volume were prepared as screening controls (‘uniformity plates’). DMSO vehicle control was added to every well of column 1, and levamisole cytotoxicity control was added at a high concentration of 10 mM in wells 1–8 and titrated 1:3, starting at a high concentration of 10 mM to 4.57 µM in wells 9–16 of column 2 of every plate. Additionally, ivermectin, levamisol and albendazole were plated as internal controls on one assay plate in 16-point, 1:2 titration down six replicate columns per compound, starting at a high concentration of 10 µM (ivermectin) or 10 mM (levamisole and albendazole). Complete details of qHTS plate controls are provided in Table S16. Assay-ready plates were sealed and frozen at −80°C until use.

### Construction of lysis vector

Lysis plasmid pλP_R_ cI-Elysis was prepared as described previously (Kwon et al., 2005), with some modifications. PhiX174 double-stranded DNA was purchased from New England BioLabs (N3023S), and the lysis *E* gene was obtained by PCR. The lysis *E* gene on PhiX174 from New England BioLabs is mutated on two sites (587 G>A, 833 G>A), with the first mutation resulting in a premature stop codon in position 7. To override the mutations, primers were designed that were longer than the ones described elsewhere (Kwon et al., 2005). Primer Egene-F was seven nucleotides longer (5′-ATGGTACGCTGGACTTTGTGGGATACC-3′) and primer Egene-R was three nucleotides longer (5′-ACATTACATCACTCCTTCCGCAC-3′). Gene *E* was amplified using Q5 High-Fidelity DNA Polymerase (New England BioLabs) with the following amplification cycle parameters: 3 min at 94°C; 32 cycles of 30 s at 94°C, 30 s at 58°C, 1 min at 72°C, with a final extension step of 7 min at 72°C. 3′ A overhangs were added using a Taq DNA polymerase plus dATP for 20 min at 72°C. The PCR product was resolved on 1% agarose gels, purified with a gel extraction kit (Qiagen) and cloned into pGEM T easy vector (Promega). The P_R_-cI857 regulatory system was obtained by PCR, as described above, using pLDR20 [American Type Culture Cell (ATCC)] as the template. The primers LPR-F (5′-CCGCGGCCCTTTAGCTGTCTTGGTTTGC-3′) and LCL-R (5′-GGGCCCGACCAGAACACCTTGCCG-3′) were described previously (Kwon et al., 2005), and contain *Sac*II and *Apa*I restriction sites (underlined in Table S12) for cloning into pGEM T easy vector.

### BG production

TOP10 *E. coli* (Thermo Fisher Scientific) transformed with pλP_R_ cI-Elysis were grown in 250 ml of Luria Broth (LB; Thermo Fisher Scientific) supplemented with ampicillin (50 μg/ml) at 28°C and 200 rpm until it reached OD_600_=0.4–0.6. Lysis *E* gene expression was then induced by incubating the culture at 42°C and 200 rpm for ∼2 h. Bacterial cell lysis was monitored periodically by measuring the OD_600_, and the BG preparation was harvested by centrifugation (10 min, 7000 rpm (3756 ***g***), 4°C) after the minimum OD_600_ of 0.1–0.3 was reached. The pellet was sequentially washed with 50 ml and 25 ml PBS, pH 7 and the final pellet was taken up in 5 ml PBS and stored at 4°C (referred to as BG concentrate). At the end of the lysis process, 1 μl culture was diluted in 50 μl LB and inoculated onto LB agar plates containing 50 μg/ml ampicillin to examine whether there were any surviving cells after 16 h at 28°C. A count of five or fewer colonies was considered a successful BG induction (Table S13).

### Calibration of COPAS biosorter for *C. elegans*

N2 *C. elegans* eggs were axenized from a mixed population of worms grown on ten NGM plates according to Stiernagle (2006). Briefly, freshly starved NGM plates were washed with 1× S basal buffer, and worms were collected and washed three times with 1× S basal buffer and resuspended in 3.5 ml water and 1.5 ml bleach (alkaline hypochlorite) solution (0.5 ml 5 N NaOH+1 ml bleach). Worms were vortexed in bleach solution for 20 s every minute for 5–10 min until worms were dissolved. Eggs were pelleted with centrifugation at 2000 rpm (939 ***g***) for 5 min and washed 3× with 1× S basal buffer. Eggs were hatched overnight in 9 ml M9 buffer (3 g KH_2_PO_4_, 6 g Na_2_HPO_4_, 5 g NaCl, 1 ml 1 M MgSO_4_, H_2_O to 1 l, sterilized by autoclaving) at 20°C with 65 rpm shaking then collected with centrifugation at 1000 rpm (235 ***g***) for 5 min. Worms were resuspended in 100 ml S medium (1 l S basal buffer, 10 ml 1 M potassium citrate, pH 6, 10 ml trace metals solution, 3 ml 1 M CaCl_2_, 3 ml 1 M MgSO_4_, components added using sterile technique; not autoclaved; IPM Scientific Inc.) with 250 mg frozen pellet of OP50. Worms were grown at 20°C with vigorous shaking, and 20 ml of worms was collected at respective time points. Early-L1s were collected at 6 h, mid-L2s were collected at 20 h, mid-L3s were collected at 28 h, mid-L4s were collected at 38 h, and adults were collected at 48 h (Sulston, 1988). Each worm harvest was loaded on a COPAS Flow Pilot (FP-500) biosorter (Union Biometrica, Inc.) in 50 ml tubes, and a bulk sort of 1000−5000 objects was performed. The COPAS was run under default settings with sheath pressure 1.70 psi (11,722 Pa), diverter pressure 2.20 psi (15,169 Pa), cleaning pressure 4.00 psi (27,580 Pa), and sample cup pressure manually adjusted between 1.20 and 1.35 psi (8274–9308 Pa) to maintain a flow rate of 15–50 events/s. Acquisition parameters were run at 500 mV signal threshold, a minimum time of flight (TOF) of 60 and extinction coefficient (EC) gain of 2.0. Gating was manually set within the experimental protocol file to collect each individual life stage based on TOF and EC. Data were collected with COPAS FP Biosorter FlowPilot software (version 1.6.0.3, Union Biometrica).

### Characterization of *E. coli* BGs as a *C. elegans* nutrient source

*E. coli* BGs and OP50 live bacteria were mixed with either DiBac4(3), a voltage-sensitive dye shown to bind selectively to depolarized membranes (Yamada et al., 2001), or 1× PBS buffer control. Concentrated TOP10 BGs (see ‘BG Production’ section) and an overnight culture of OP50 live bacteria (OD600>0.9) were divided into two 500 μl aliquots. Next, 0.5 μl of 1 mM DiBAC4(3) dye and 1× PBS were added to respective aliquots of each bacterial preparation. Bacterial solutions were incubated for 30 min at ambient temperature on a RotoFlex Plus tube rotator (Argos Technologies) set at a device revolution speed of 15. NGM plates were coated with 200 μl of either *E. coli* BGs or OP50 live bacteria with or without DiBAC4(3). N2 *C. elegans* were expanded to each of the four NGM plates with sterile chunking so that each plate received 40−60 worms. Worms were grown at ambient temperature, protected from light for 5 days until plates were cleared of BGs or bacteria. Worms were collected from respective plates, washed and resuspended in 10 ml 1× S basal buffer. *C. elegans* were sorted and counted by life stage with the COPAS biosorter calibration protocol above, and each life stage from the different bacterial treatments was further sorted based on GFP intensity and TOF. COPAS fluorescence settings were run with a 488 nm laser at 40.0 mW power, 1.5 green gain and 320 V photomultiplier tube (PMT). TOP10 BGs without dye treatment were used as control for basal GFP signal for each stage of development, and total numbers of worms per life stage (with and without GFP fluorescence) were counted across all growth media. Worms were plated with the COPAS into a 384-well uClear microtiter plate, and representative worms were imaged on a GE InCell analyzer 2200 (GE Healthcare). The plate was imaged as one field per well with a Nikon 10×/0.45 NA Plan Apo objective, CFI/60. Excitation wavelengths (brightfield and FITC) were selected in Quad 1 with respective emission wavelengths Cy3 and FITC imaged with 2D deconvolution image processing. The laser autofocus used an offset of 0.0 with 0.100 s exposure and an offset of 16.0 with 0.02 s exposure, respectively, and 1×1 binning was used in a horizontal serpentine acquisition motion to collect images of each well. Fig. S11 further describes the experimental design.

### Assay development employing LSC and BG nutrient source

*C. elegans* strain PE254 ubiquitously express cytoplasmic GFP throughout development; expression is detectable during late embryogenesis, through all larval stages and in adults (Lagido et al., 2008). Strain PE254 worms were collected from eight NGM plates as above. The mixed population of *C. elegans* was resuspended in 30 ml 1× S basal buffer and plated at ten GFP worms per well of each life stage into respective wells of 384-well uClear microtiter plates with the COPAS biosorter run with the calibration protocol and settings from above. *E. coli* BGs diluted to OD_600_=0.6 or OP50 live bacteria diluted to OD_600_=0.05, 0.10, 0.15 or 0.2 in S medium were added to respective plates at 30 μl per well manually with a Capp pipette (BioVentures, Inc.). Next, 300 nl levamisole, ivermectin or albendazole was transferred to respective columns of assay plates in an eight-point 1:3 titration in duplicate, starting at a high stock concentration of 10 mM, 10 µM or 10 mM, respectively, with a Mosquito HTS nanoliter liquid handler (STP Labtech). DMSO and 50 µM final concentration levamisole were added to designated columns as vehicle and cytotoxicity controls, respectively. See Table S14 for additional details. Plates were covered with a Breath-Easy seal (Thermo Fisher Scientific) and weighted metal lids with gas exchange pores (Fujifilm Wako Automation Corp.). Worms were grown in the presence of compound or DMSO for 7 days at 21°C and 65 rpm in an Incu-Shaker (Benchmark Scientific). Worms were imaged every 24–72 h on an Acumen eX3 laser scanning cytometer (STP Labtech). Plates were read with default reader settings, with scan 1 488 nm laser, green (FL-2) detection channel, 400 voltage, sensitivity of 2 and triggered in the green PMT. Green objects detected by the reader were identified as worms based on the filtered FL-2 object parameters perimeter (100-2200), area (1000-180,000), and peak intensity (150-1500). Viability was monitored as an increase in GFP fluorescence quantified as total number and total object area (µm^2^) of FL-2 filtered objects per well. LSC data were collected using cellista 4 (version 4.3.4.0, STP Labtech). More detail can be found in Table S15.

### Primary qHTS

*C. elegans* strain PE254 were collected from 12 NGM plates as above, transferred to 250 ml S medium supplemented with OP50 *E. coli* pellet collected from 2 l overnight culture, and grown for 5 days at 21°C with vigorous shaking in an Incu-Shaker Mini Shaking Incubator (Benchmark Scientific, Inc.). Eggs were axenized as above and hatched overnight in M9 buffer at 21°C and shaken at 65 rpm. Assay ready plates were thawed and allowed to equilibrate to ambient temperature. BGs in S medium (30 µl/well) at an OD_600_=0.85 were manually added across plates with a Capp pipette. Synchronized L1 worms from above were collected, diluted and plated at ten worms/30 µl/well with a Capp pipette into assay-ready microtiter plates pre-plated with BGs and compounds (240-fold dilution). Plates were read on an Acumen eX3 LSC as above, with resolution settings of X=2 µm and Y=16 µm within 4 h of plating and at 3- and 7-day time points (see Table S15 for further detail). No ethical approval or guidance is needed for working with nematodes.

Mammalian cell toxicity was evaluated in HEK293 cells (ATCC) using ATP levels as an indicator of metabolically active cells. Assays were performed in 1536-well plates (Greiner white/solid bottom, high base), and luminescence measurements were made on a ViewLux (Perkin Elmer) (Martinez et al., 2021; Solinski et al., 2019). Assay-ready plates in 1536-well format were prepared as above using the 384-well Echo-compatible source plates; 25 nl of a seven-point titration with 1:5 dilution starting at a high concentration of 2.5–20 mM of compound was added to respective wells with an Echo 555 acoustic dispenser. A titration of digitonin, 20 mM to 610 nM in DMSO (83.3 µM to 2.5 nM final assay concentration), was used as an intraplate cell toxicity control titration. DMSO vehicle and 83.3 μM digitonin final concentration (64 or 32 replicate wells per assay plate, respectively) were used to establish the assay signal window. Plates were stored at −80°C until use and equilibrated to ambient temperature prior to cell dispense. Then, 1500 cells were added at 6 μl assay volume/well (240-fold dilution) across plates with a Multidrop Combi dispenser (Thermo Fisher Scientific) in standard Dulbecco's modified Eagle medium. Cell viability-dependent luminescence levels were read following the addition of 4 μl CellTiter-Glo (Promega) after 24 h incubation with the test compound. See Table S16 for further details.

HEK293 cells in this study were sent to Johns Hopkins Genetic Core Resource Facility in Baltimore, MD, for identity confirmation by short tandem repeat profiling. Cell cultures were routinely tested for Mycoplasma contamination using a MycoAlert PLUS Mycoplasma Detection Kit (Lonza Bioscience, LT07) according to the manufacturer’s protocol. Cells in this study tested negative for Mycoplasma.

### Data analysis for assay development and qHTS

Assay statistics including signal-to-background ratio (Eqn 1), signal-to-noise ratio (Eqn 2) and Z′ (Eqn 3) (Southall et al., 2009; Zhang et al., 1999) were calculated for each life stage of *C. elegans* strain PE254 grown on various bacterial media treated with the intra-plate control compounds, 50 µM levamisole toxicity control and DMSO neutral control. Concentration–response curves for average total filtered FL-2 object area (‘GFP area’) and total filtered FL-2 number of objects (‘number of worms’) for each respective compound were fit with the nonlinear regression log (agonist) versus response–variable slope (four parameters) algorithm in Prism (GraphPad Software). Hillslope and/or EC_50_ constraints were applied as needed to all life stages per compound across the 7-day time course, and EC_50_ values were calculated.
(1)

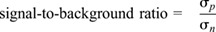

(2)

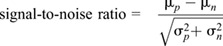

(3)

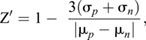
where σ is the mean signal and µ is the standard deviation for the treated (*p*) or neutral (*n*) controls.

Data from the Anti-Infectives screen were normalized by plate to corresponding intra-plate controls as previously described (Inglese et al., 2006). Assay statistics were calculated using the same respective controls. Total object ‘GFP’ area (µm^2^) and number of worms were normalized to the average signal of DMSO-treated wells as zero percent neutral control, and the average signal of 41.7 µM levamisole-treated wells as −100% activity. In-house software was used to fit the resulting inter-plate titration data to the standard Hill equation, and concentration–response curves were classified by activity as previously described (Southall et al., 2009). Curve fits assignments of −1.1, −1.2, −2.1, −2.2 and −3 were considered active and visually confirmed. These data were refit in Prism (version 8.1.2) with nonlinear regression log(agonist) versus response–variable slope (four parameters) fit (Eqn 4).
(4)

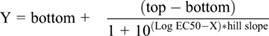


Three-axis plots of qHTS data for *C. elegans* and HEK293 cells in Fig. 2C,E, respectively, were plotted using a custom software called qHTSWaterfall (Queme et al., 2022 preprint). For Self-Organizing Map (SOM) analysis small molecules with a defined structure in the library (885) were clustered based on structural similarity (729-bit ToxPrint chemotype fingerprints, v2.0_r711, https://toxprint.org/) using the SOM algorithm yielding 187 clusters. Each cluster was evaluated for its enrichment of active compounds (by comparing the IC50s of compounds in the cluster to the library average) at day 3 and day 7 using Student’s *t*-test.

### Follow-up confirmation testing and *C. elegans* life-stage analysis

*C. elegans* strain PE254 were collected from six NGM plates and washed. Their eggs were axenized as above, hatched overnight in M9 buffer at 21°C and shaken at 65 rpm. Assay-ready plates were prepared as above with an 11-point, 1:3 titration of 96 compounds identified as active from the qHTS screen based on curve classification and maximum response. Next, 30 μl/well BGs in S medium at an OD_600_=0.86 and ten worms/30 μl/well of synchronized L1 were manually added across plates with a Capp pipette, and plates were read on an Acumen eX3 LSC as above within 1 h of plating and every 24 h for a 7-day time course (Table S17). Data were normalized as above, and concentration–response curves were fit in Prism with nonlinear regression log(agonist) versus response–variable slope (four parameters) fit.

After 7 days of compound treatment in six-well culture plates seeded with a mixed population of 2000 *C. elegans* strain PE254, a life-stage distribution analysis was determined by TOF and EC using the COPAS FP BioSorter, followed by bulk collection in a 50 ml conical tube for use in proteomics studies.

*C. elegans* collected into 50 ml tubes per treatment were further consolidated into 15 ml tubes with centrifugation. Pelleted worms were frozen in ∼100 µl residual buffer at −80°C for 30 min, then resuspended in 300 μl NP40-lysis buffer (Thermo Fisher Scientific) containing protease inhibitor cocktail (Sigma-Aldrich) and transferred to a mortar containing LN2, ground to fine white powder and allowed to thaw on ice. Worm extract was transferred to a 1.5 ml Eppendorf tube and centrifuged at 13,200 rpm (16,100 ***g***) for 15 min at 4°C. Supernatant was removed and transferred to a new 1.5 Eppendorf tube, and an aliquot of protein was diluted 1:5 in lysis buffer for protein quantification with standard bicinchoninic acid (BCA) assay (Thermo Fisher Scientific). See Table S18 for further details.

### Proteomics sample preparation

Compound and control treatment groups comprising three biological replicates each were prepared for proteomic digestion by trypsin prior to HPLC-MS/MS analysis. The groups consisted of untreated PBS and DMSO control groups and bortezomib (4 µM)-, trametinib (1 µM)-, LY411575 (500 nM)-, selumetinib (10 µM)- and selinexor (20 µM)-treated groups. Around 30 µg of the proteins estimated from the above BCA results for each sample was added to RapidGest (0.1% w/v) and TCEP (20 mM), followed by reduction incubation (55°C, 850 rpm, 20 min). After reduction incubation, the samples were centrifuged, and iodoacetic acid (IAA) was then added to each sample (15 mM) for incubation (30 min, dark environment). Chilled acetonitrile (−20°C) was then added to each sample in a 9:1 v/v ratio. The samples were immediately incubated on ice and then centrifuged (4°C, 18,000 ***g***, 10 min). As much acetonitrile as possible was then removed without disturbing the precipitated protein pellet. The samples were then evaporated on the Centrivap Complete Vacuum Concentrator for 10 min. A volume of 30 µl 0.02 mg/ml trypsin was added to each sample and transferred to a thermal mixer for overnight incubation (37°C, 850 rpm, 15-18 h). Formic acid was added to each sample to quench the reaction prior to HPLC-MS/MS analysis.

### HPLC-MS/MS analysis

All proteomic HPLC-MS/MS analysis was performed using an UltiMate 3000-nano LC system coupled to a Orbitrap Fusion Lumos Tribrid mass spectrometer equipped with a Nanospray Flex ion source (Thermo Fisher Scientific). Peptides were loaded onto the trap column (Acclaim PepMap 100 C18, 75 µm×2 cm, particle size 3 µm, 100 Å) and separated with an analytical column with the spray tip (75 µm×30 cm, 1.7 µm, 100 Å) (CoAnn Technologies) using a 200 min method (∼180 min gradient). Peptides were loaded onto the trap column by autosampler using loading solvent (2% acetonitrile in 98% UHPLC-grade water) at a flow rate of 4 µl/min. Elution of peptides from the analytical column was performed using a 180 min gradient starting at 98% A (0.1% formic acid in UHPLC-grade water) at a flow rate of 250 nl/min. The mobile phase was maintained at 2% B (80% acetonitrile, 19.9% water, 0.1% formic acid) for 5 min, 2-9% B for 4 min, 9-38% B for 141 min, 38-50% B for 25 min, 50-90% B for 3 min, and maintained at 90% B for 10 min, followed by re-equilibration of the column with 2% B for 10 min. Column oven parameters were set at a temperature of 40°C.

The OrbitrapFusion™ Lumos™ Tribrid™ mass spectrometer was operated in positive-ionization mode with the Easy-Max NG ion source with spray voltage set at 1800 V and ion transfer tube temperature set at 250°C. The MS scan was operated in data-dependent acquisition mode, with full MS scans over a mass range of 375-1800 m/z with detection in the Orbitrap (120 K resolution) and with auto gain control (AGC) set to 1.0×10^6^. The fragment ion spectra were acquired in Orbitrap (15 K resolution) with a normalized collision energy of 28% in HCD activation mode. In each cycle of data-dependent acquisition analysis, the most intense ions were selected for the MS/MS analysis, and the cycle time for MS and MS/MS analysis was set as 2 s. The AGC for MS/MS was standard (instrument default), and maximum injection time was 22 ms. Precursor ions with charges of +2 to +7 were isolated for MS/MS sequencing. The MS/MS isolation window was 1.2 Da, and the dynamic exclusion time was set at 60 s (after one MS/MS acquisition) with a mass tolerance of ±10 ppm.

### Data analysis for proteomics

Proteome Discoverer software suite (v2.4, Thermo Fisher Scientific) with Sequest algorithm were used for peptide identification and quantitation. The MS raw data were searched against a Swiss-Prot *C. elegans* database (version August 2020, reviewed database) consisting of 4165 entries using the following parameters: precursor ion mass tolerance of 10 ppm and a fragment ion mass tolerance of 0.02 Da. Peptides were searched using fully tryptic cleavage constraints, and up to two internal cleavages sites were allowed for tryptic digestion. Fixed modifications consisted of carbamidomethylation of cysteine. Variable modifications considered were oxidation of methionine residues and N-terminal protein acetylation. Peptide identification false discovery rates were limited to a maximum of 0.01 using identifications from a concatenated database from the non-decoy and the decoy databases. Label-free quantification analysis used the ‘Precursor Ions Quantifier’ node from Proteome Discoverer and normalized by total peptide amount. The hierarchical clustering (heat maps) in Fig. 4 was generated using the embedded program within Proteome Discoverer 2.4. with recommended settings. Network diagrams were created in STRINGdb.

## Supplementary Material

10.1242/dmm.049863_sup1 Supplementary information
