# Supplementary material for: In vivo quantitative high-throughput screening for drug discovery and comparative toxicology
Source: Dis Model Mech. 2023 Mar 20;16(3):dmm049863. doi: 10.1242/dmm.049863 (PMC10067442; doi:10.1242/dmm.049863)
Supplement: Supplementary information [file dmm-16-049863-s1.pdf]

Figure S1

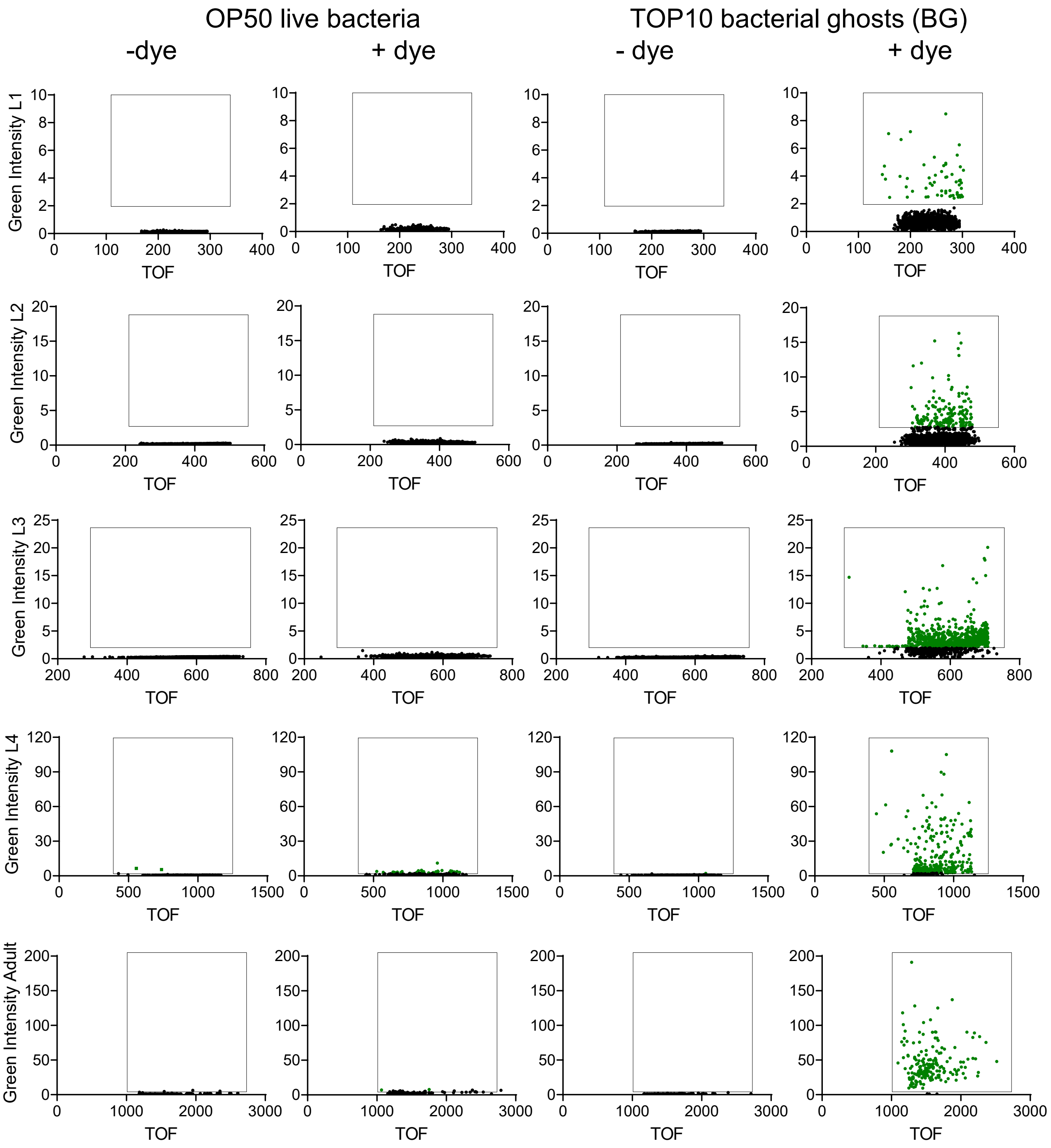

Fig. S1. Individual scatter plots of strain N2 *C. elegans* life stage-COPAS biosorted worms grown for five days on OP50 live bacteria (left) or TOP10 ghost (right) with or without DiBAC4(3) dye. Worms were subsequently sorted by green fluorescence intensity within each life stage with gating manually set based on TOP10 ghost capsules without DiBAC4(3) dye as the negative control. Green fluorescing worms are shown as green symbols and non-green worms are shown as black symbols for L1, L2, L3, L4 and adults from top to bottom, respectively.

Disease Models & Mechanisms • Supplementary information

Figure S2

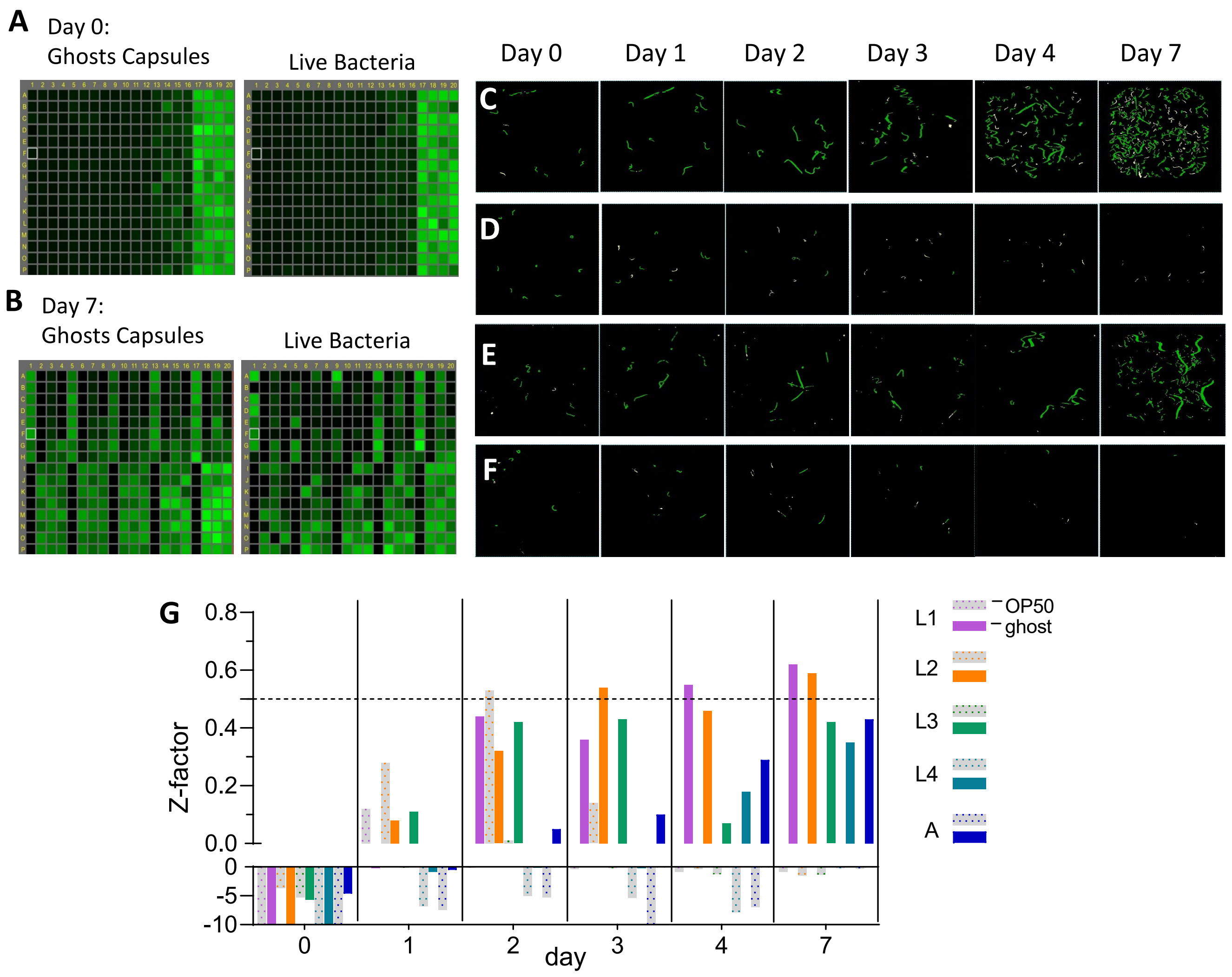

Fig. S2. **Acumen eX3 laser scanning cytometer plate images from worms grown on ghost capsules vs OP50 live bacteria.** Ghost capsules (*A and B, left*) plated at an OD600 of 0.65 compared OP50 live bacteria (*A and B, right*) plated at an OD600 of 0.2 at day 0 (**A**) immediately after plating or (**B**) day 7. Plate images are auto contrasted based on total area of gated GFP objects where black is low total area and bright green is high total area. Worms were plated as L1 in columns 1-4, L2 in columns 5-8, L3 in columns 9-12, L4 in columns 13-16, and adults in columns 17-20 with the COPAS biosorter. Compound treatment is as described in **Supplementary Methods**. Representative well images from Acumen laser scanning cytometer for each day of time course for *C. elegans* grown on ghost capsules (**C** and **D**) or OP50 live bacteria (**E** and **F**) and treated with 0.5% DMSO (**C** and **E**) or 50 uM levamisole (**D** and **F**). Objects identified as GFP expressing *C. elegans* based on size and fluorescence intensity are shown in green while objects excluded from analysis are shown in white. (**G**) Z-factor life-stage analysis.

Disease Models & Mechanisms • Supplementary information

Figure S3

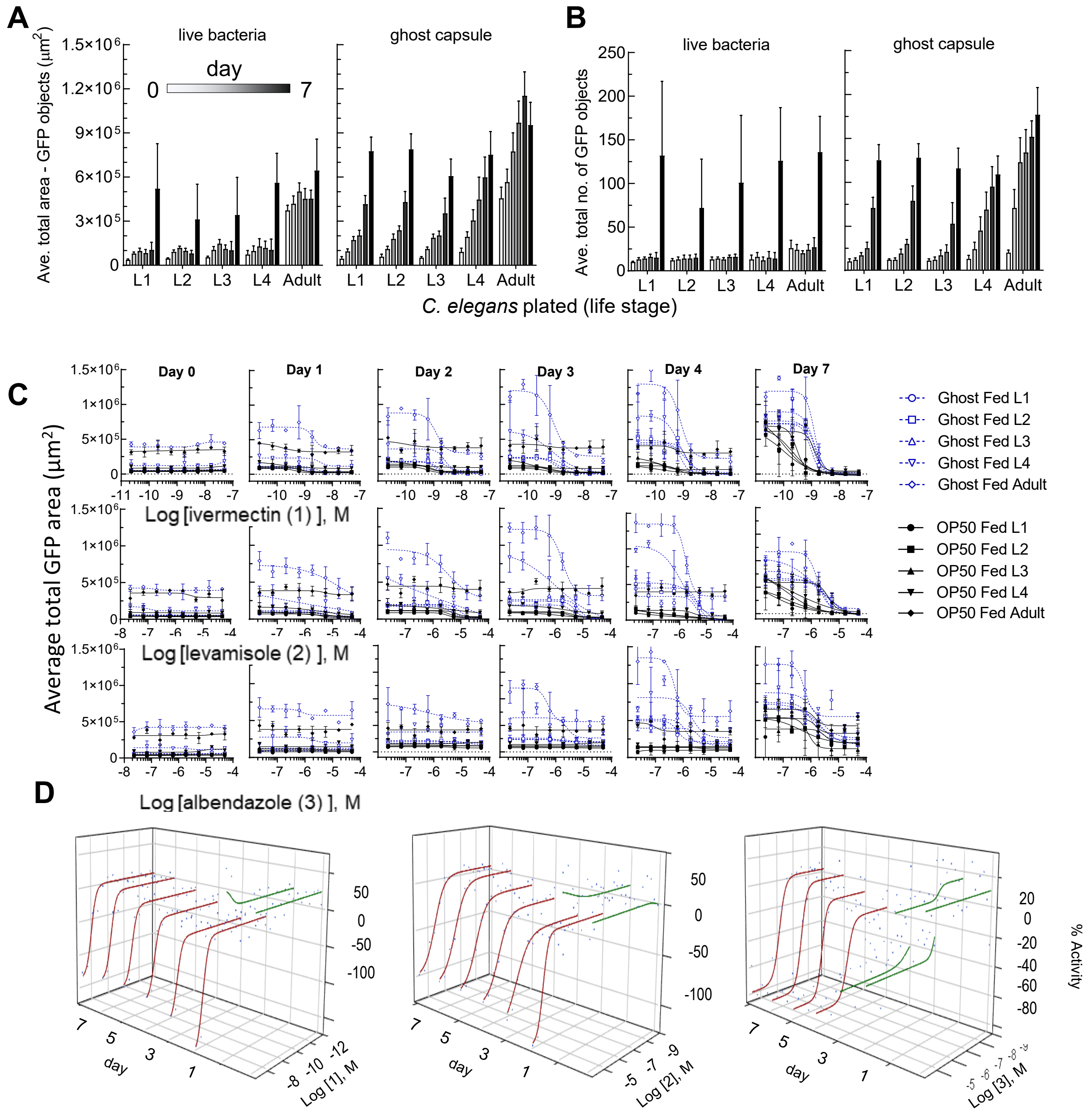

Fig. S3. Comparison of PE254 *C. elegans* total GFP area vs total number of GFP objects across life stages grown on OP50 live bacteria or BGs. (**A**) Total area (μm^2) of GFP objects vs (**B**) total number of GFP objects measured for each life stage treated with 0.5% DMSO grown for 7-days on OP50 live bacteria or BGs. Error bars represent s.d. of 8 replicate wells. **(C)** CRCs across a PE254 *C. elegans* 7-day time course at respective life stages treated with ivermectin (**top**), levamisole (**middle**), or albendazole (**bottom**) grown on OP50 live bacteria (OD_{600}=0.20, black solid symbols) or BGs (OD_{600}=0.65, blue open symbols). Responses measured as ave. total area of GFP objects (μm^2), error bars represent the s.d. of two duplicate wells. Constraints of hill slope < -3 and log EC_{50} > -10 set for ivermectin curves except those on day 0, and a constraint of Hill slope < -3 was applied to all albendazole and levamisole curves except day 0. (**D**) 3-axis DRC plots for GFP worm area for anthelmintic controls measured daily for a 7-day time course. Inhibitory curves, red, and data from days where no activity was observed, green.

Figure S4

A

Parameters	Assay Parameter	
	CellTiter-Glo	
1536-well plates	7	
Compounds tested	739	
Points per titration	7	
Data points	5,173	
Signal-to-background	26.0 ±0.4	
Output Signal	DMSO<br>1232.5 ±112,4	Digitonin<br>46.7 ±7.4
CV	9.2 ±1.3	16.0 ±1.3
Z' factor (Median)	0.776 ± 0.05	
Control condition 1	83 μM Digitonin	

B

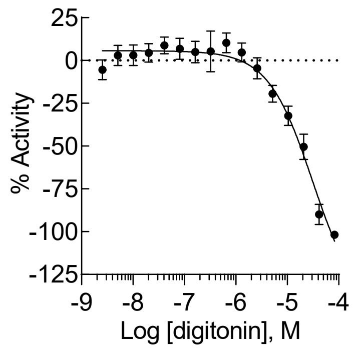

Fig. S4. **Mammalian cellular toxicity of anti-infectives library.** (**A**) Assay performance metrics.(**B**) CRC and respective EC_{50} (31.3 μM) for the digitonin screening control at 24 h post-treatment. Error bars represent the standard deviation of 14 replicate wells.

Figure S5

Parameters	Assay Parameter	
	GFP Area – Day 7	# Worms – Day 7
384-well plates	3 (+1 DMSO)	
Compounds tested	96	
Points per titration	11	
Data points	1,056	
Signal-to-background	385.1 ± 207.0	103.3 ± 21.0
Output Signal	9.37E5 ±7.18E4	332.7 ± 30.2
CV	7.6 ±1.4	9.1 ± 1.9
Z' factor (Median)	0.76 ± 0.04	0.70 ± 0.05
Control condition 1	41.7 μM Levamisole	

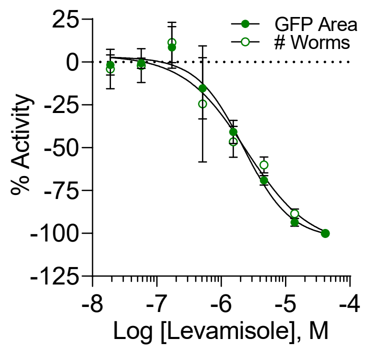

Fig. S5. **Re-test summary statistics.** (**A**) Re-testing information and assay performance metrics across the two output parameters measured on the Acumen laser cytometer based on GFP intensity of the worms, GFP area and worm number, measured on day 7 of the screening time course. (**B**) CRCs and respective EC_{50} values (using GFP area EC_{50} =2.23 μM; using #worms EC_{50} =2.45 μM) for the levamisole screening control at day 7. Error bars represent the standard deviation of 4 replicate wells.

Figure S6

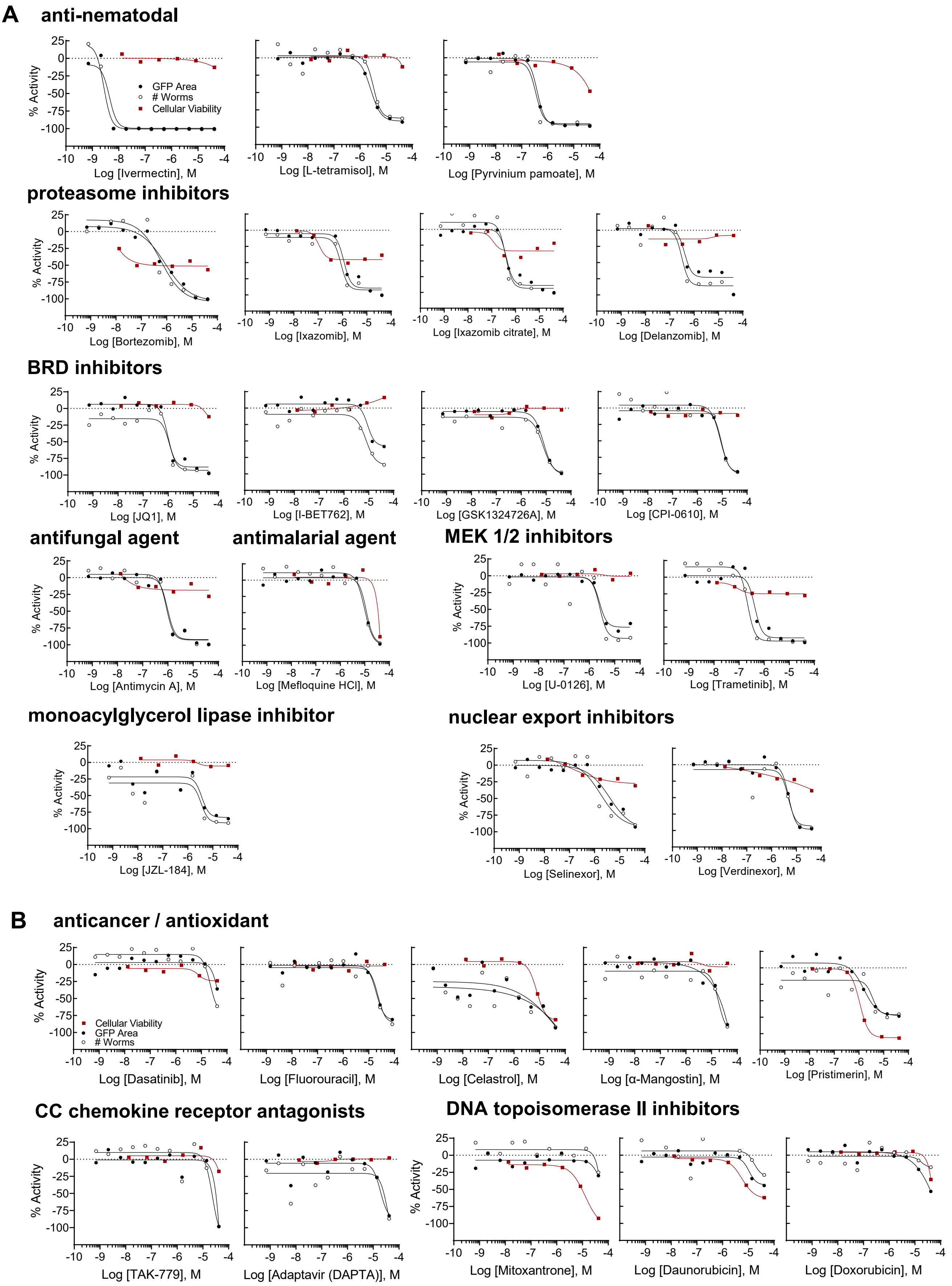

Disease Models & Mechanisms • Supplementary information

Figure S6 (continued)

C antibacterial / antimicrobial

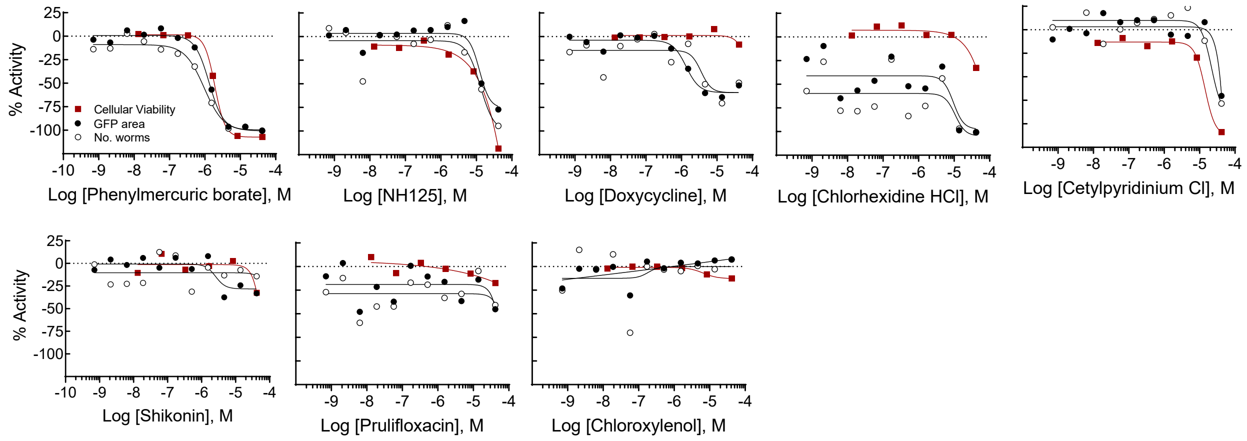

antiviral

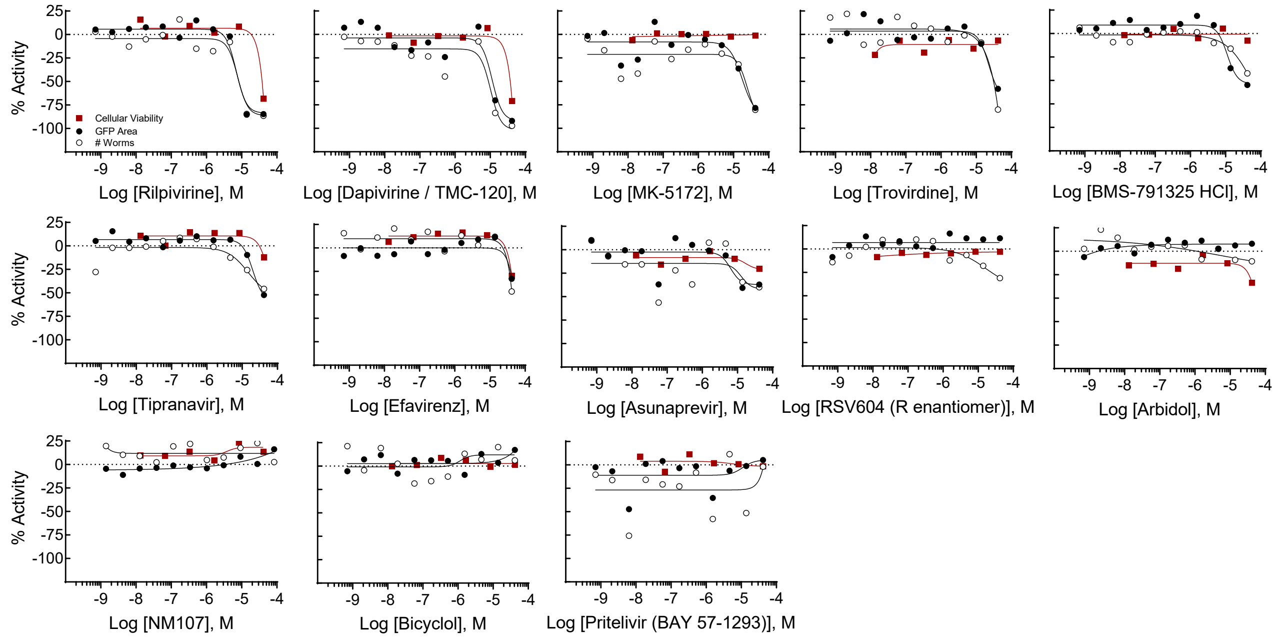

γ-secretase inhibitors

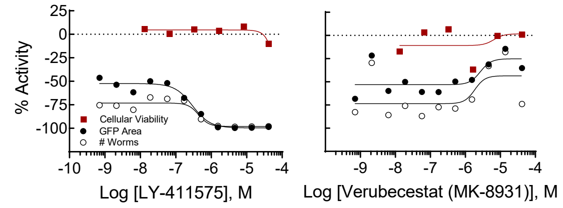

apoptosis activators

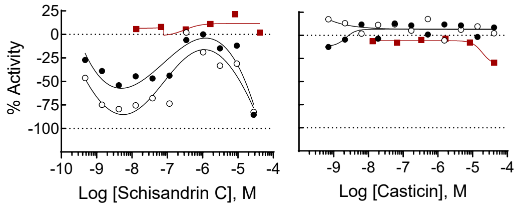

deubiquitinase inhibitors

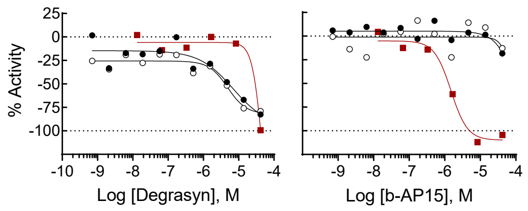

Disease Models & Mechanisms • Supplementary information

Figure S6 (continued)

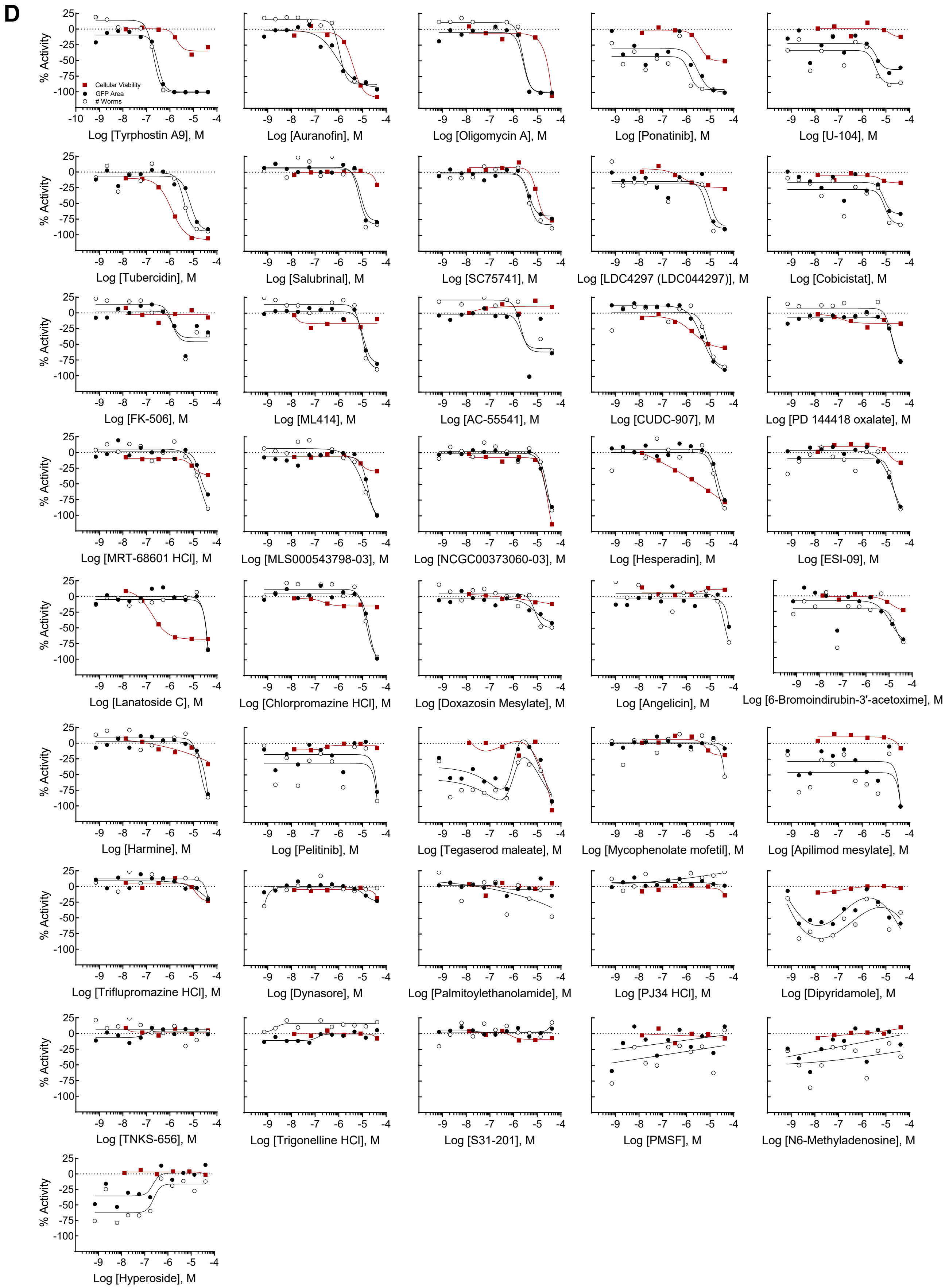

Disease Models & Mechanisms • Supplementary information

Fig. S6. Re-test 11-pt. dose response curves (DRCs) for compound classes displaying effects on *C. elegans* viability in the primary qHTS.

A. Antiparasitic agents with reported anti-nematodal properties (3/3); proteasome inhibitors (4/10); BRD inhibitors (4/6); the single antifungal agent; antimalarial agents (1/4); MEK 1/2 inhibitors (2/3); monoacylglycerol lipase (MAGL) inhibitors (2/2); and nuclear export inhibitors (2/2) in the anti-infectives library demonstrating reproducible effects on *C. elegans* viability.
B. Compounds with anticancer/antioxidant properties (5/14); C-C chemokine receptor (CCR) antagonists (2/5); and DNA topoisomerase II inhibitors (3/5) in the anti-infectives library demonstrating reproducible effects on *C. elegans* viability.
C. DRCs for compound classes with primary qHTS toxic effects on *C. elegans* that only partially reconfirmed activity in follow-up analysis. Eight antibacterial/antimicrobial compounds selected for follow-up from the 14 contained in the anti-infectives library, 5 reconfirmed with high quality inhibition curve classes; 13 antiviral compounds selected for follow-up from the 25 contained in the anti-infectives library, 8 demonstrated high quality inhibition curve classes with various potencies and efficacies; the γ-secretase inhibitor selected for follow-up from the six inhibitors contained in the collection reconfirmed similar activity (note that the low basal signal obtained at low concentrations of the compound was due to a plating artifact where the center of the plate demonstrated lower signal than the outer wells), and the β-secretase inhibitor selected for follow-up from the five inhibitors contained in the library did not reconfirm activity (the moderate increase in signal is also most likely due to the plating artifact above); two deubiquitinase inhibitors from the three inhibitors contained in the anti-infectives library, one reconfirmed good activity while the other demonstrated minimal to no activity in follow-up analysis; and two apoptosis activators of the five activators contained in the library, one reconfirmed moderate low potency activity, while the second was inactive in follow-up analysis.
D. DRCs of compounds with individual annotated MOAs selected for follow-up based on primary qHTS toxic effects on *C. elegans*.

The two output parameters measured on the Acumen laser cytometer based on worm GFP intensity, GFP area (solid black circles) and worm number (open black circles), measured on day 7 of the screening time course are plotted as black lines where data was normalized to 41.7 μM levamisole screening control as -100% activity. Mammalian cellular toxicity response (red squares) are plotted for each compound where data is normalized to 83 μM digitonin cytotoxicity control as -100% activity. Fraction indicates number of active vs. total number of compounds in the anti-infective library annotated with a similar MOA. Curves were fit in GraphPad Prism.

Disease Models & Mechanisms • Supplementary information

Figure S7

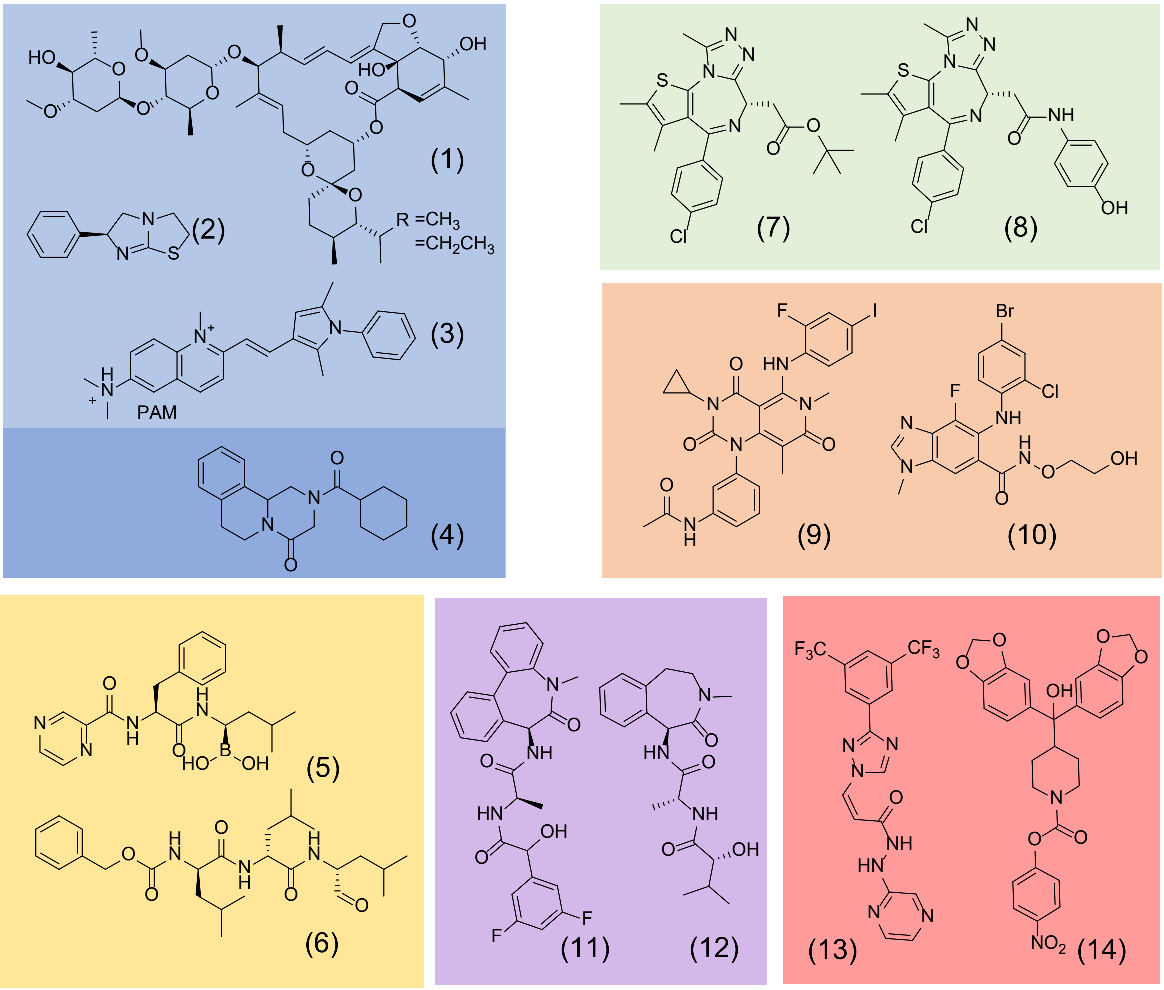

Fig. S7. Compounds selected for evaluation on impact of life-stage viability described in main text Figure 3c. 1-4, antihelminth agents; 5-6, proteasome inhibitors; 7-8, bromodomain inhibitors; 9-10, MEK inhibitors; 11-12, γ-secretase inhibitors; 13, nuclear protein export inhibitor, 14, monoacylglycerol lipase inhibitor. 1, Ivermectin; 2, Tetramisol; 3, Pyrvinium pamoate; 4, Praziquantel; 5, Bortezomib; 6, MG-132; 7, JQ1; 8, OTX-015; 9, Trametinib; 10, Selumetinib; 11, LY-411575; 12, Semagacestat; 13, Selinexor; 14, JZL-184.

Figure S8

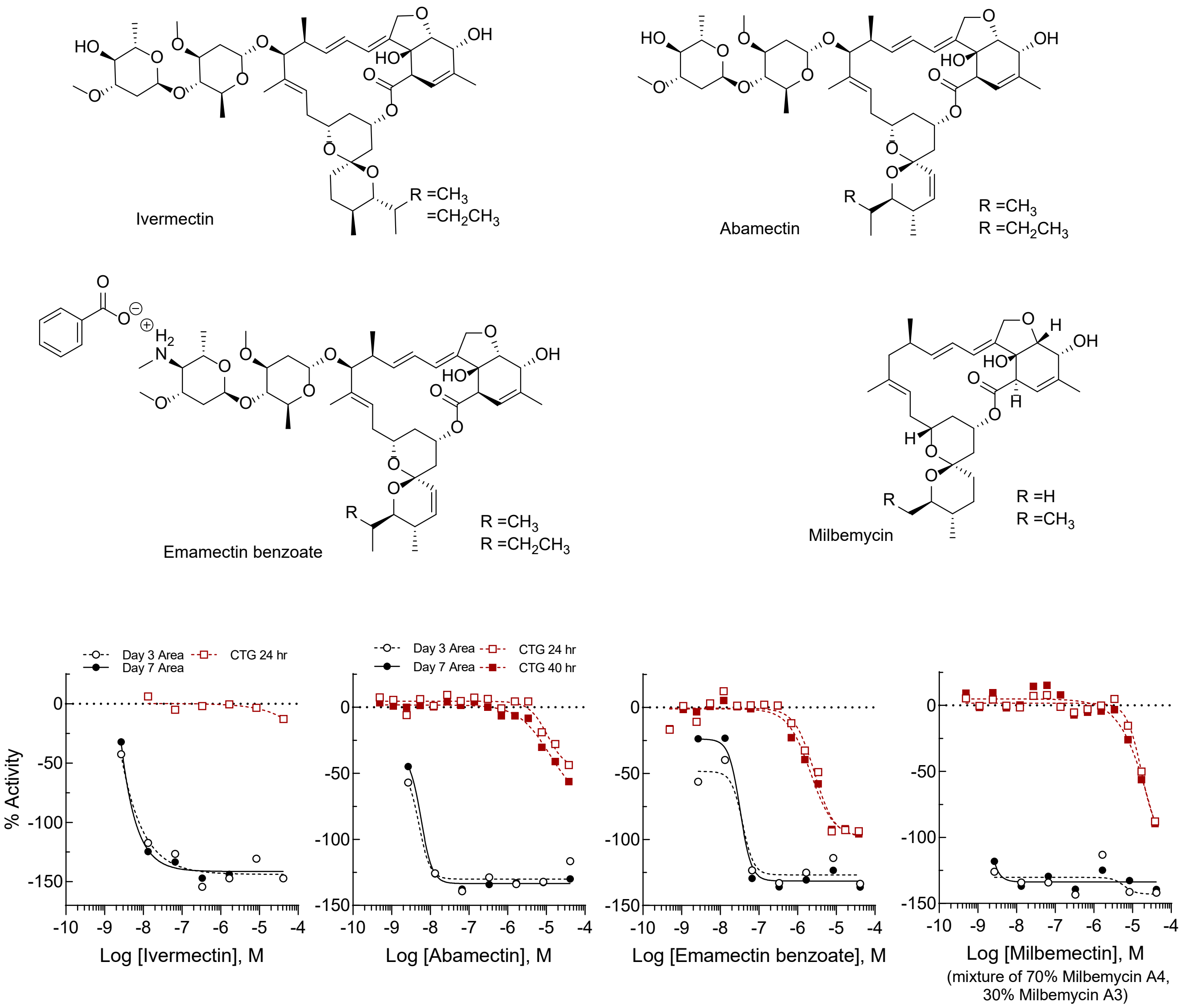

Fig. S8. Chemical structures and associated dose-response data from the Tox21 library qHTS for Ivermectin analogs. Emamectin benzoate (MK-0244) is a 4”-deoxy-4”-methylamino derivative avermectin B1a/b (Epi-methylamino-4′′-deoxy-avermectin). Milbemycin lacks the α-l-oleandrosyl-α-l-oleandrosyl moiety found in the avermectins. Dose-response curves for activity on *C. elegans* (black symbols) and HEK293 cells (red symbols) for days and times, respectively, indicated in the legend.

Disease Models & Mechanisms • Supplementary information

Figure S9

A

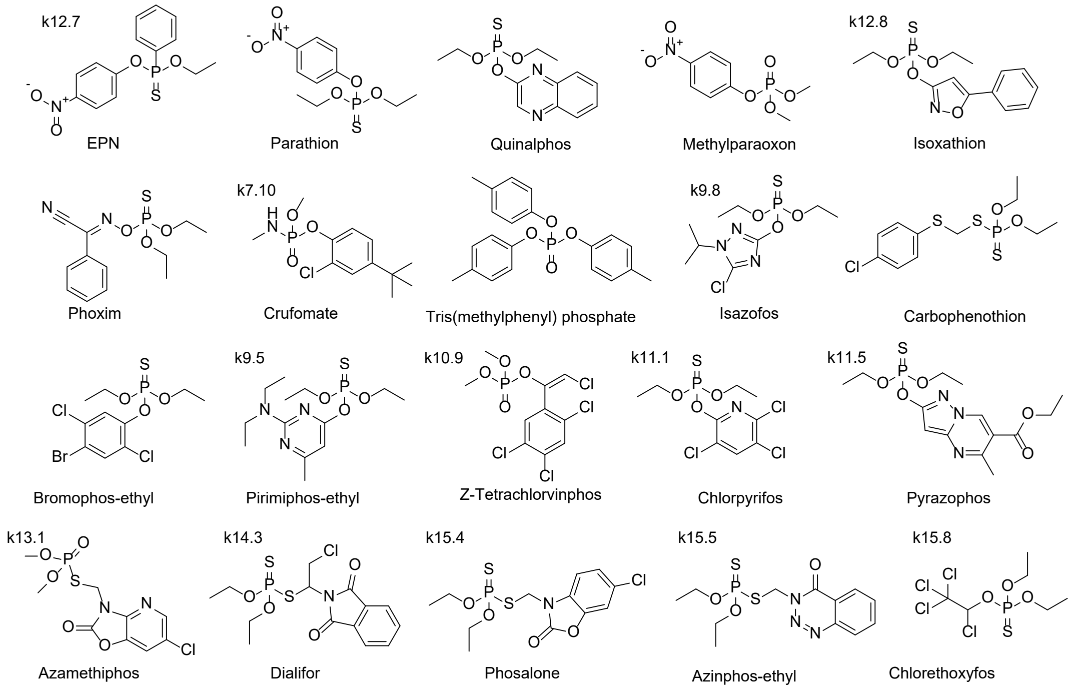

B

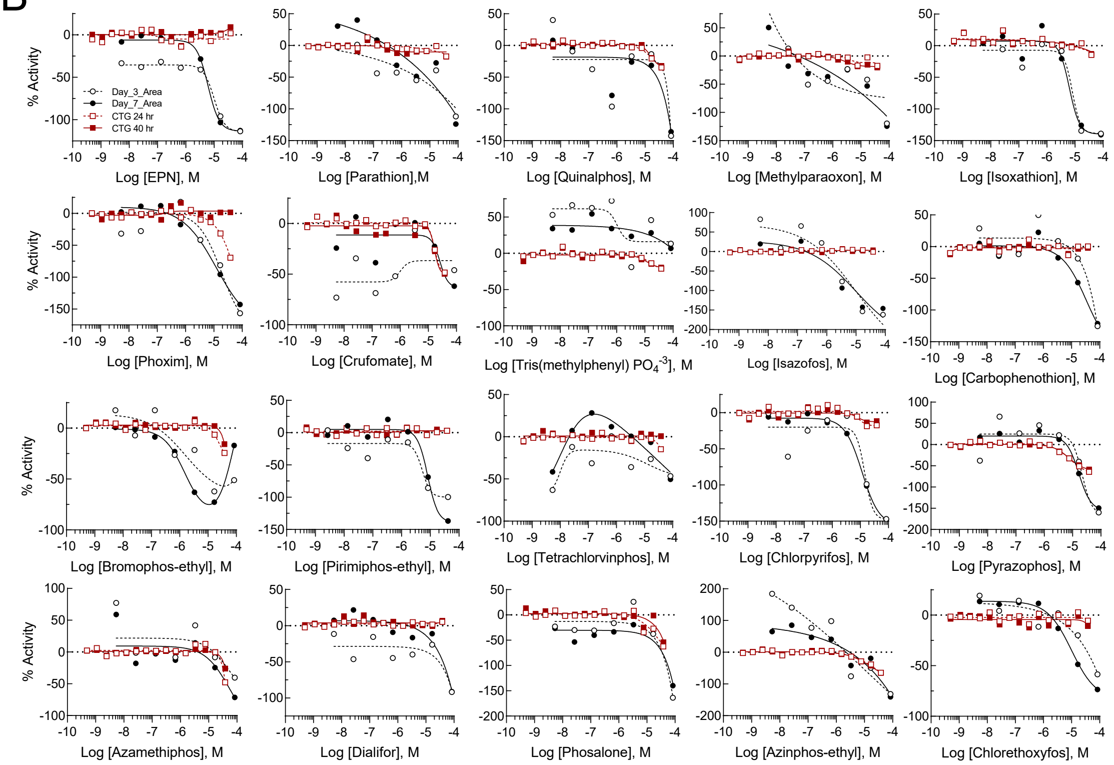

Fig. S9. Chemical structures and associated dose-response data from the Tox21 library qHTS for organophosphates. (**A**) Chemical structures of organophosphates, thiophosphates and the phosphonothioate (EPN) and associated *k*-clusters. (**B**) Dose-response curves for activity on *C. elegnas* (black symbols) and HEK293 cells (red symbols) for days and times, respectively, indicated in the legend.

Figure S10

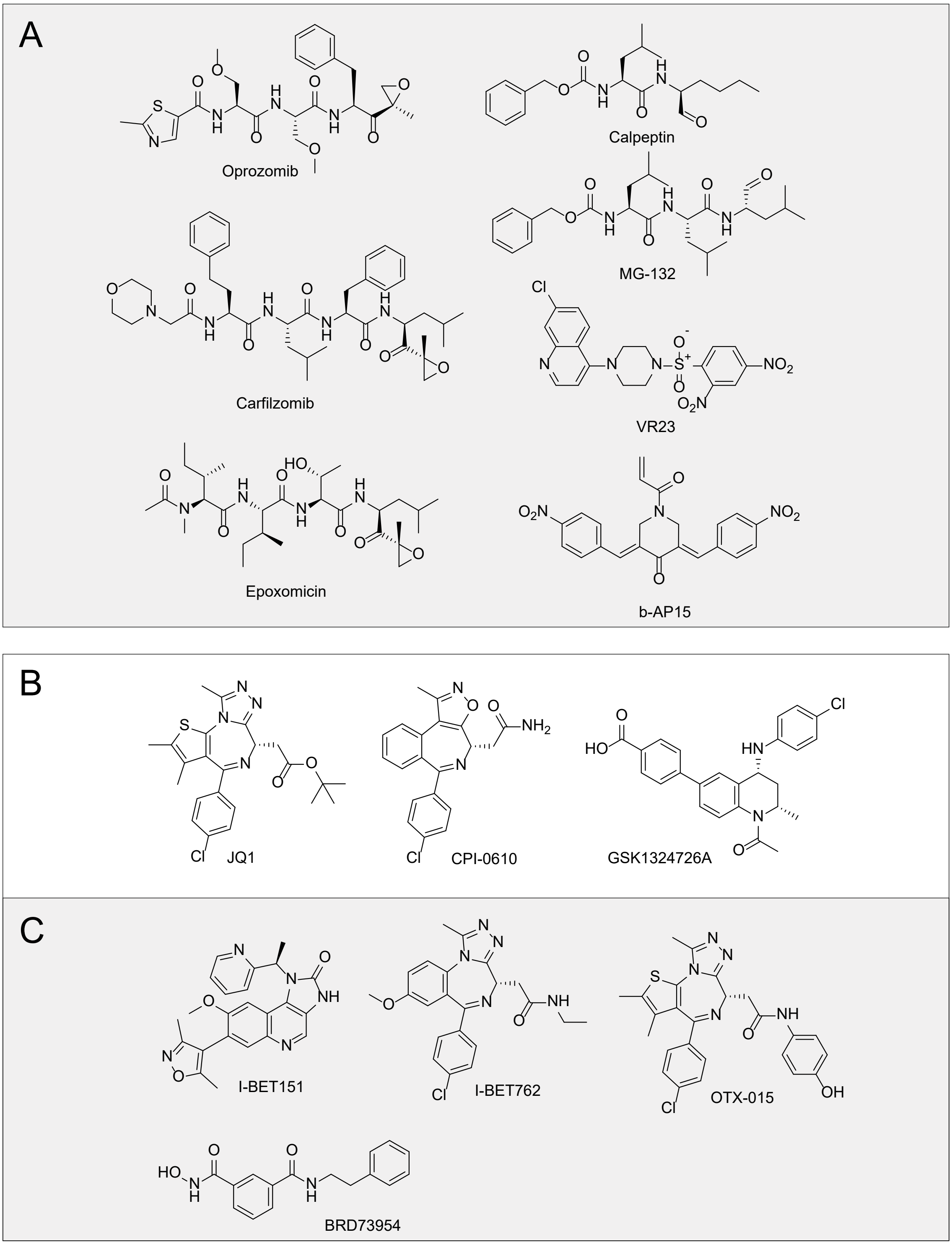

Fig. S10. Proteasome and bromodomain inhibitor structures. (**A**) Inactive non-boronic acid proteasome inhibitors. (**B**) Active and (**C**) inactive bromodomain inhibitors.

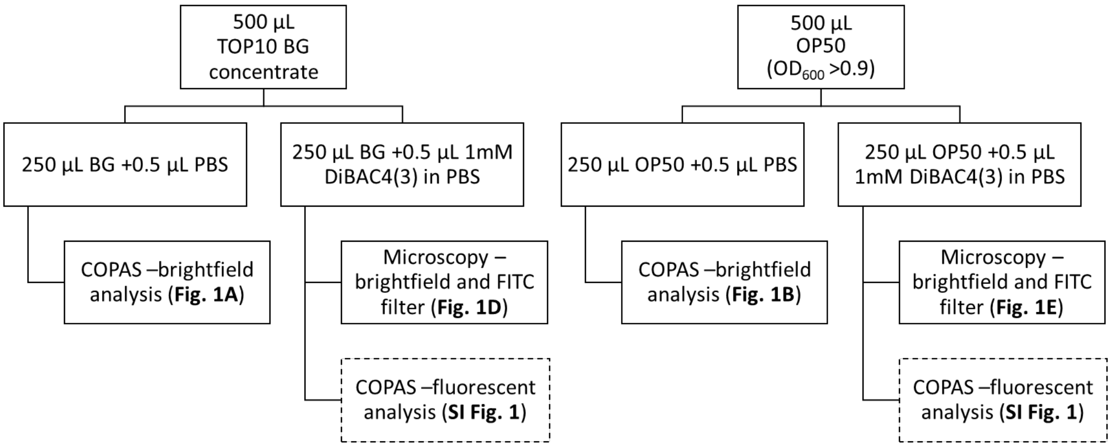

Fig. S11. Workflow for characterization of *E. coli* BGs as a *C. elegans* nutrient source.

Table S1. ssay statistics for GFP area (μm^2) of C. elegans grown on OP50 live bacteria or TOP10 BGs as nutrient source over 7 day time course

Click here to download Table S1

Table S2. Control agent EC50 and max response values based on GFP area (μm^2) of *C. elegans* grown on OP50 live bacteria or TOP10 bacterial ghosts

Click here to download Table S2

Table S3. Anti-Infectives compund collection primary qHTS summary table

Click here to download Table S3

Table S4. Compounds selected from Anti-Infectives collection for follow-up

Click here to download Table S4

Table S5. Anti-Infectives follow-up screen IC50 and max response summary table

Click here to download Table S5

Table S6. Proteomics analysis across 7 groups of compound treatments summary table

Click here to download Table S6

Table S7. Proteomics analysis for Bortezomib treated samples

Click here to download Table S7

Table S8. Proteomics analysis for Bortezomib treated samples

Click here to download Table S8

Table S9. Tox21 sub-library annotated with MOA information

Click here to download Table S9

Table S10. TOX21 sub-library primary qHTS summary table and associated Tox21 data for caspase 3/7, MitoTox, SBE, hERG and viability assays

Click here to download Table S10

Table S11. Comparison of Lowest Effective Concentrations (LECs) of 34 compounds from Tox21 ATP (LUM) and larval growth (EXT) assays with C. elegans qHTS

Click here to download Table S11

Table S12. Construction of E. coli lysis vector

Click here to download Table S12

Table S13. Preparation of E. coli bacterial ghosts (BGs)

Click here to download Table S13

Table S14. Life-stage sorted C. elegans strain PE254 384-well plate viablity assay

Click here to download Table S14

Table S15. PE254-GFP-FLuc C. elegans 384-well plate library qHTS

Click here to download Table S15

Table S16. HEK293 mammalian cell CellTiter-Glo 1536-well plate viability library qHTS

Click here to download Table S16

Table S17. PE254-GFP-FLuc C. elegans 384-well plate follow-up qHTS

Click here to download Table S17

Table S18. Life-stage analysis and preparation of whole worm protein extracts

Click here to download Table S18
